# Oral nicotine pouches and public health: evidence from an updated scoping review

**DOI:** 10.1093/ntr/ntag203

**Published:** 2026-09-06

**Authors:** Nargiz Travis, Kenneth E. Warner, Jamie Hartmann-Boyce, Maciej L. Goniewicz, Claire Ma, Rafael Meza, David T. Levy

**Affiliations:** 1 Lombardi Comprehensive Cancer Center, Georgetown Medical University, Washington, DC 20007, United States; 2 Department of Health Management and Policy, School of Public Health, University of Michigan, Ann Arbor, MI 48109, United States; 3 Department of Health Promotion and Policy, University of Massachusetts, Amherst, MA 01003, United States; 4 Department of Health Behavior, Roswell Park Comprehensive Cancer Center, Buffalo, NY 14203, United States; 5 Rutgers Cancer Institute, Rutgers University, New Brunswick, NJ 08901, United States; 6 Department of Population Health Sciences, BC Cancer Research Institute, Vancouver, BC V5Z1L3, Canada; 7 School of Population and Public Health, University of British Columbia, Vancouver, BC V6T1Z3, Canada

**Keywords:** oral nicotine pouch, nicotine dependence, oral nicotine products, public health, addiction, scoping review

## Abstract

**Introduction:**

The popularity of oral nicotine pouches (ONPs) is rising in the US and worldwide. This review provides an update on their potential public health impact.

**Methods:**

We searched six electronic databases for ONP-related empirical studies through September 30, 2025, updating an earlier review. Outcomes included ONP product characteristics, use patterns, beliefs, perceptions, toxicity, sales, and marketing.

**Results:**

We identified 73 new studies published since our previous review (majority conducted in the US; 12 industry-funded). Data from heterogeneous national surveillance sources indicate a slow but steady increase in current ONP use prevalence among youth in 2024, with the National Youth Tobacco Survey reporting 1.8% prevalence among middle and high school students and the Monitoring the Future study reporting 2.6% prevalence among 10th and 12th graders. Current use among adults was relatively low in 2022–2023 (0.4%–0.8%), with higher use among ages 18-44 and current tobacco users. US sales have more than tripled between 2021 and 2024. Marketing strategies increasingly emphasize potentially youth-appealing claims, with online and social media as key promotional channels. ONPs generally contain a higher proportion of freebase nicotine than other oral nicotine products and tobacco-containing pouches, with nicotine delivery increasing proportionally with nicotine strength. Nicotine uptake from higher strength ONPs (≥6 mg) typically exceeds that from cigarettes in adult smokers, while ONPs consistently score lower in positive subjective ratings than cigarettes. Preliminary evidence from short-term, heterogeneous studies suggests that ONP use can reduce smoking in daily smokers, generally resulting in dual use rather than complete cigarette cessation. ONPs are considerably less toxic than cigarettes, but their long-term health impacts remain understudied.

**Conclusions:**

The review underscores the need for further research on ONP health effects, their efficacy in long-term smoking cessation, and broader public health impact in tobacco-naïve populations.

**Implications:**

This updated review synthesizes the rapidly expanding evidence base on ONPs, providing timely, policy-relevant insights to inform tobacco regulatory bodies as they evaluate ONP product standards, marketing practices, and population-level public health impacts.

## Introduction

The popularity of ONPs continues to increase worldwide, with commercial market intelligence reports estimating the global market value at nearly US$2 billion in 2022 and projecting it to reach US$23 billion by 2030.^1^ Leading ONP manufacturers report increasing sales, mainly in the US, with an expanding geographic reach into new markets, such as Pakistan and Saudi Arabia.^2–4^ Globally, the regulatory landscape of ONPs is constantly evolving, with many countries still developing legislative proposals.^5^ While ONPs are commercially available without restrictions in many countries (e.g., Pakistan, United Kingdom [UK]), others either banned (e.g., the Netherlands, Belgium, France), limited ONP sales to pharmacies (e.g., New Zealand, Australia, Canada), or restricted sales of high-dose nicotine strength (e.g., Finland).^5,6^ In the US, while many ONP products are commercially available, only 20 ZYN products (ten non-tobacco-flavored pouches of 3 mg and 6 mg nicotine) and 6 on! products (tobacco, mint, and citrus flavors of 6 mg and 9 mg nicotine) have been authorized for marketing by the Food and Drug Administration (FDA) as of 2025.^7,8^ These authorizations were granted under the Premarket Tobacco Product Application (PMTA) pathway, with the FDA determining that marketing these products is “appropriate for the protection of public health”. This shifting regulatory framework fundamentally reshapes the public health calculus. It places ONPs within a tobacco harm reduction policy matrix, while simultaneously tying legal market presence to post-market surveillance and marketing restrictions designed to prevent youth uptake. However, marketing authorizations do not equate to a Modified Risk Tobacco Product order, which requires a separate, distinct authorization to allow for explicit reduced-risk or reduced-exposure claims to the public.

Our first scoping review summarized initial empirical evidence to assess the potential role of ONPs in harm reduction and to identify unintended consequences of their use (e.g., youth uptake, tobacco-naïve initiation, dual use).^9^ It included studies published through January 2024 that investigated ONP product characteristics, toxicity, use prevalence estimates, product appeal, risk perceptions, sales trends, and industry marketing tactics. The review concluded that ONPs appear less toxic than cigarettes and may deliver comparable nicotine, presenting an alternative for combustible product users, though most evidence came from industry-funded studies. Furthermore, the review found a high prevalence of ONP awareness (35%–42%) and susceptibility to use (9-44%) among US youth and young adults.^9^ These findings, along with the increasing sales of ONPs in the past year reported by industry,^3,4,10^ may indicate further growth in ONP use. As research on ONPs is fast-moving, the objective of the present work is to update our previous scoping review, focusing on the same research questions and incorporating newer evidence, while broadening the list of databases to include trial registries.

## Materials and methods

This study represents a formal update to our previously published scoping review on ONPs. To preserve comparative integrity and systematically track the rapidly evolving ONP landscape, the core research questions and the five primary outcome domains—Use Patterns, Beliefs and Perceptions, Product Characteristics, Toxicity, and Marketing and Sales—remain identical to the baseline study. While the baseline review restricted its search to three major electronic indexing engines through January 10, 2024, this update expands the field to six comprehensive databases through September 30, 2025. The strategic addition of three specialized trial and registry databases was implemented to capture emerging human experimental data, pharmacokinetic profiles, and actual use studies that recently emerged or may have been missed during the initial review iteration. This review adhered to the guidelines for the Preferred Reporting Items for Scoping Reviews (PRISMA-ScR). The preregistered protocol is available on Open Science Framework.^11,12^

### Search strategy

We used search terms described in our first review.^9^ In addition to MEDLINE, EMBASE, and Web of Science, we included three new clinical trial databases: CENTRAL, clinicaltrials.gov, and the WHO ICTRP. Our updated searches were conducted on September 30, 2025, with searches in MEDLINE, EMBASE, and Web of Science limited to publications from 2023 to exclude evidence already identified in the original review. No date restrictions were applied to the three newly added clinical trial databases, as they were not included in the baseline search of the original review. Searching them from their respective dates of inception was methodologically required to establish a complete historical baseline for clinical protocols and published results. Identified records were combined and deduplicated. Four reviewers (NT, CM, JLB, NL) independently screened titles and abstracts using Covidence (Veritas Health Innovation, Melbourne, Australia). Two reviewers (NT, CM) independently screened full-text articles for eligibility criteria. One reviewer (NT) searched the references of eligible papers for additional sources due to resource constraints and the volume of the mapped literature. Disagreements were resolved by consensus. The complete PubMed search strategy is presented in Supplementary File S1.

### Eligibility

We included peer-reviewed empirical studies on ONPs within the five outcome categories, using the same eligibility criteria described in our earlier review.^9^ Full texts in English and German were eligible for inclusion. We planned to include a list of eligible studies in other languages as an appendix; however, the full-text eligibility screening revealed no such studies. We excluded studies on non-nicotine pouches (e.g., nicotine analogue pouches) and studies that investigate a wider range of oral nicotine products (e.g., pouches, lozenges, gum) without reporting ONP-specific findings. We excluded literature reviews, editorials, case reports, commentaries, letters, and conference proceedings.

### Data extraction

Two reviewers (NT, CM) independently extracted key data from the full texts of eligible studies using a standardized data extraction tool in Microsoft Excel (Microsoft Corporation, 2019). Data entries were compared, and any disagreements were resolved by consensus.

### Data synthesis

We synthesized data and presented findings in tables and narration, grouped by outcome categories and, within those, by specific outcomes. In studies investigating use patterns, we considered both current (often defined as past-30-day use or use every day/-some days/occasionally) and ever (lifetime) use of ONPs. Definitions of these outcomes across studies are further described in Supplementary Table S2. Our secondary analysis stratifies findings by population (youth and adults, history of tobacco/nicotine use), country, and source of funding (industry vs. non-industry). This review update focuses on new evidence since our first scoping review. Supplementary Tables S1–S4 include studies from both reviews, and the Discussion explicitly assesses whether new evidence reinforces, extends, or contradicts conclusions from the earlier review.

## Results

A total of 481 unique titles and abstracts were screened for eligibility, with 181 assessed in full text. Seventy-three studies were eligible for inclusion (including 12 industry-funded) (Fig. 1). Supplementary Table S1 presents characteristics of the included studies from both scoping reviews, comprising 135 studies.

### Use patterns of ONPs

Thirty-seven new studies reported on use patterns of ONPs, focused on the US,^13^ Europe,^10^ Asia,^6^ Canada,^2^ and Australia.^1^ Five received tobacco industry funding (BAT, Altria).^14–18^ Supplementary Table S2 summarizes studies on ONP use prevalence.

#### US prevalence estimates

Among nationally representative youth surveys, the Population Assessment of Tobacco and Health (PATH) survey data yielded estimated lifetime (current) ONP use prevalence of 0.9% (0.2%) among adolescents ages 12-17 in 2022-2023.^13^ The National Youth Tobacco Survey (NYTS) indicated an increase in lifetime (current) ONP use from 1.9% (0.8%) to 3.5% (1.8%) among middle and high school students between 2021 and 2024.^19–22^ Monitoring the Future survey indicated an increase in lifetime (current) ONP use among 10^th^ and 12^th^ grade students from 3% (1.3%) in 2023 to 5.4% (2.6%) in 2024, with most reporting dual use with e-cigarettes.^23^ Among adolescent current ONP users in 2024, 29% reported frequent use, and 22% reported daily use.^20,21^

In nationally representative surveys of adults in 2022-2023, PATH survey yielded estimates of lifetime (current) use of 3.3% (0.8%),^13, 24^ and Tobacco Use Supplement to the Current Population Survey (TUS-CPS) yielded estimates of lifetime (current) use of 2.7% (0.4%).^25–27^

#### Prevalence estimates in other countries

Lifetime (current) ONP use was estimated at 3.3% (1.2%) among British youth in 2024,^28^ 3.7% (2.6%) among adolescents in Quebec, Canada in 2024,^29^ 26% (19%) among youth and adults in Australia in 2023,^30^ 5.4% among German youth in 2022-2023,^31^ and 17.2% (9.4%) among Pakistani youth and adults in 2022^32^ (with current use at 3.4% [2.7%] among adolescent boys [girls] in 2023-2024).^32^ In Finland, lifetime (current) use was 14.4% (3.7%) among youth and adults in 2022,^33^ and 17.2% (7.2%) among youth in 2023.^34^

Among adults, the prevalence of lifetime (current) ONP use in 2024 was 13% in the UK,^35^ and 5.4% (1%) in Great Britain.^28^ In England, current use was 0.4% in 2023.^36^ Lifetime (current) use was 9.2% (4.3%) in Poland in 2024.^37^ In Saudi Arabia, lifetime use was14.2% among the general population^38^ and 21.2% among those with tobacco use history in 2024/2025.^39^ In Canada, lifetime (current) use was 27% (12%) among adult college students in 2024.^40^ Current use was estimated at 1.4% in Sweden and 0.8% in Denmark in 2019.^15^

#### ONP consumption behaviors

Current ONP users reported keeping the pouch in their mouth for ≤20 minutes,^14, 16, 30^ (except for ≤60 minutes in Sweden^14^), using them frequently or daily,^21, 32, 40^ or multiple times a day.^41^ The average consumption of pouches per day, often reported in categories, was 5–6 in some samples of ONP users in the US,^16,17^ ≤5 in Germany and Switzerland,^14^ between 2–6 in Saudi Arabia,^39,42^ 6–8 in Denmark,^14,15^ and 8 in Sweden.^14,15^

In the US, the most commonly used brand among youth and young adults (ages 13–24) was ZYN, whereas both ZYN and Lyft (Velo) were commonly used among adults ages 25–40.^21,43^

The most commonly used ONP flavor in the US, Germany, Switzerland, Sweden, and Denmark was mint, both among youth^19, 21^ and adults.^14,25,26^ Both mint/menthol and fruit flavors were commonly used among Australian youth and adult users.^30^

The most frequently reported nicotine strength used, often reported in categories, was 4 mg in a sample of ONP users in the US,^16^ 6–15 mg in Germany and Switzerland,^14^ 11–15 mg in Sweden and Denmark,^14^ ≤ 4 mg in Finland,^33^ 5 mg in Australia,^30^ 6–10 mg in Saudi Arabia,^42^ and 4–7 mg in Canada.^40^

#### Demographic characteristics of ONP users

Supplementary Table S3 presents studies on demographic characteristics of ONP users. Among adolescents, ten new studies from the US, Germany, Finland, Canada (Quebec), Pakistan, and the UK found a higher likelihood of lifetime ONP use in males,^13,20,23,28,31^ older ages,^13,20,23,31^ American Indians/Alaska Natives (AI/AN),^20^ non-Hispanic white,^13,20,23^ multicultural race,^20^ rural residents,^23^ current,^13,28^ former^13,28^ and lifetime^19^ smokers, current,^13,23,28^ former,^13^ and lifetime^19^ e-cigarette users, current,^13^ former^13^ and lifetime^19^ smokeless tobacco (SLT) users, current tobacco/nicotine product users,^31^ past-year other oral tobacco^13^ and cannabis users,^13,28^ and those reporting higher sensation seeking^31^ and lower subjective social status.^31^ Studies of adolescent current user demographics were limited by small sample sizes, but suggest higher use in males,^13,20,23,29,34,44^ older ages,^13,20,23,29^ AI/AN and non-Hispanic white,^13, 20, 23^ Hispanic,^45^ current^13, 19, 28, 29, 34^ and former^13^ smokers, current^13, 19, 34^ and former^13^ SLT users, current vapers,^13,19,23,28,29,34^ current multi-product users,^44^ and past-year other oral tobacco^13^ and cannabis users.^13, 28^

In adults, twelve new studies from the US, Australia, Canada, Poland, Pakistan, Saudi Arabia, and Great Britain found higher odds of lifetime ONP use among ages 18–25,^41^ 18–30,^39^ 18–34,^26,28^ 18–39,^38^ 18–60,^37^ and 18–64,^13,25^ males^13,25–28,30,38–40^ (except no association with gender in one Polish study^37^), and non-Hispanic whites^13, 25–27, 40^ (except no association with ethnicity in one British study^28^). Similarly, current ONP use was more likely among adults ages 18–24,^24^ 18–60,^37^ 18–64,^13,25^ males,^13,24–27,30^ and non-Hispanic whites.^13,24–27^

Higher lifetime ONP use was associated with current,^13,25,27,28, 30,37,38^ former,^13,25,27,28,30^ and lifetime^40^ smoking, current,^13,27, 28,30,38^ former,^13, 27, 28^ and lifetime^37, 40^ e-cigarette use, current,^13^ former,^13^ and lifetime^27^ SLT use, past-year use of other oral tobacco^13^ and cannabis use,^13,40^ quit intentions,^25^ and past-year smoking quit attempts.^25^ Higher current ONP use was associated with current^13,27,28,30,37,41^ and former^13, 27, 30^ smoking, current,^13,24,27,28,30,37^ former,^13,27^ and lifetime^37^ e-cigarette use, current,^13,24^ former,^13^ and lifetime^27^ SLT use, and past-year smoking quit attempts^25^ and cannabis use.^28^

While active employment was associated with higher lifetime and current ONP use,^26,37^ associations with education level were mixed (ranging from no association^13,28,39^ to both positive^26^ and negative^25^ associations). Household income and financial status were not associated with ONP use,^13,24,25,37,40^ except in one US study where income of ≥$75 000 was associated with higher ONP use.^26^

#### Impact on other tobacco/nicotine product use

Eleven studies (three industry-funded; Altria and British American Tobacco [BAT]) focused on the association between ONP and other tobacco/nicotine product use.

##### Non-industry-funded studies

In an 8-week-long three-arm randomized control trial (RCT, n = 33), daily smokers with low socioeconomic status and no intention to quit were assigned to either continue smoking, use nicotine-containing e-cigarettes, or use 4 mg ONPs instead of cigarettes. By week 8, only one individual completely switched from smoking to ONPs, while three individuals stopped using ONPs, and the majority remained dual ONP/cigarette users. ONP use decreased the baseline average cigarettes per day (CPD) of 15 cigarettes by nearly half.^46^ In another 4-week RCT (n = 30), adult daily smokers with no intention to quit were assigned to either 3 mg or 6 mg ONP use and instructed to switch to ONPs completely. Both ONP strengths reduced CPD, while only 6 mg promoted complete smoking cessation in two participants.^47^ A prospective study of Californian adolescents who reported past 6-month e-cigarette use found that current ONP use was associated with a lower likelihood of continued vaping and lower vaping frequency at 6-month follow-up.^45^

In cross-sectional studies, having quit cigarettes was associated with daily ONP use in US adults, while having quit e-cigarettes was associated with current non-daily, but not daily, ONP use.^27^ Among daily or occasional English smokers attempting to quit smoking, ONPs were used in 3.1% of quit attempts and showed no association with quit success.^48^ Among Pakistani current adult ONP users (most using ONPs >one year), 73% reported dual use with cigarettes while also reporting they would like help quitting ONPs.^41^ Youth and adult dual ONP/cigarette users reported no difference in CPD before and after starting ONP use.^32^ In contrast, 41% of Saudi Arabian adults with a history of tobacco use who tried ONPs reported successful smoking cessation with ONPs;38% reported a reduction in smoking.^39^

##### Industry-funded studies

A 6-month parallel-group RCT (n = 100) of adult smokers with no intention to quit assigned participants to either an ad libitum use of 2, 4, and 8 mg mint-flavored ONPs of their own choice with instructions to discontinue smoking, or to a control group of continued smoking. Over 90% reduction in CPD was reported in the ONP group at6 months compared to baseline.^17^ In an observational study, adult smokers and /or SLT users with no intention to quit used ad libitum ONPs of various flavors and nicotine strength (1.5-8 mg) in a 5-day trial, followed by continued at-home use.^16^ At 6 weeks, 27% of smokers and 71% of SLT users reported no cigarette/SLT use, while reporting continued use of ONPs. Among those who continued smoking or SLT use, a 50%-99% reduction in those products used per day was reported by 39% of smokers and 14% of SLT users, compared to baseline. A wider variety of ONP flavors was associated with fewer CPD, independent of nicotine level.^16^ In another observational study, young adult daily cigarette smokers used 4 mg ONP (mint and citrus flavors) ad libitum for 6 weeks.^18^ While only 3% of smokers reported no cigarette use by week 6, 82% reported a reduction in CPD: 48% reduced CPD by 1%–30%, 18% by 30%–50%, and 16% by more than 50%.

#### Reasons for ONP use

Surveys of Pakistani adults identified peer influence,^41^ curiosity to try,^32^ recreational use,^32^ ease of use in tobacco-restricted areas,^41^ and quitting smoking^32^ as the main reasons for ONP use. Among Australian adolescents and young adults, common reasons for ONP use were the availability of flavors,^30^ pleasant taste, and attractive packaging,^30^ sport performance enhancement,^49^ in nightlife and party settings,^49^ help with nicotine cravings, and vaping and smoking reduction or cessation.^30,49^

### Beliefs and perceptions

#### Product awareness

##### US

NYTS survey data show that 41% of adolescents reported awareness of ONPs in 2022.^19^ Among adults, 45% reported awareness of ONPs in 2021, mainly from stores, the internet/social media, direct mail/ email, and print media ads. Younger, female, Black, and Hispanic (vs. white) adults showed lower odds of ONP awareness.^50^ In a 2021–2022 young adult sample, 20% reported awareness after briefly viewing a Velo ONP ad.^51^

##### Other countries

In 2024, 41% of British youth ages 11–18 and between 55%–71% of adults had heard of ONPs.^28,35^ In Poland, 24% of adults were aware of ONPs, with higher awareness among current smokers and lifetime e-cigarette or heated tobacco product (HTP) users.^37^ Among Australian youth and adults, 77% reported awareness, with higher odds in men and current tobacco and e-cigarette users.^30^ Among Saudi Arabian adults, 59% reported awareness, higher among males and those ages 18-39.^38^ In 2022, 77% of Pakistani youth and adults were aware of ONPs.^32,41^

#### General opinions

Findings for a localized qualitative study of a small sample of adult current smokers in North Carolina indicated that participants perceived ONP discreteness, flavors, and packaging as the main appealing factors to youth and young adults, while also perceiving ONPs as a product to supplement rather than replace smoking.^52^

##### Product interest/susceptibility

In the US, ONP-related health concerns negatively impacted the interest in trying ONPs in adult current smokers.^52^ Lower education (high-school diploma or less) was positively correlated with intentions to try ONPs among young adults.^51^

Nearly 21% of Pakistani tobacco-naïve adolescents were susceptible (defined as the absence of a firm decision not to use ONPs in the coming year and accepting these from friends) to trying ONPs, with 15% intending to try them within a year.^44^ In a sample of Australian adolescents and younger adults who were aware of ONPs but had never used them, 70% were susceptible to trying them. Susceptibility (defined as the absence of a firm decision regarding curiosity about ONPs, accepting these from friends, and intentions to use them in the next 6 months) was driven by having positive attitudes towards ONPs, lower addictiveness perceptions, and current or former use of e-cigarettes, but not tobacco.^53^ Among Saudi Arabian adults, nearly 60% were susceptible to trying ONPs (defined as the absence of a firm decision regarding curiosity about ONPs, willingness to use if offered by a friend or readily available, and having limited knowledge of ONP health risks).^38^

#### Risk perception

Among US adolescents and younger adults, cigarettes were perceived as most harmful, followed by nicotine e-cigarettes, ONPs, nicotine lozenges, tablets, gum, THC e-cigarettes, toothpicks, patches, and non-nicotine e-cigarettes.^54^ Nearly 25% of UK adults perceived ONPs (ZYN) as harmful; 57% perceived them to be less harmful than cigarettes.^35^ In Poland, 61% of adults perceived ONPs as harmful as cigarettes.^37^ Among Pakistani youth and adults, 27% perceived ONPs as equally harmful, 31% as more harmful, and 42% as less harmful than cigarettes.^32^

Current ONP users were more likely to have low harm perceptions of ONPs,^54,55^ and to perceive them as less harmful than cigarettes.^37,41^ In a BAT-funded survey, 34% of adult regular ONP users in Germany, 34% in Switzerland, 37% in Denmark, and 8% in Sweden perceived ONPs as harmful as cigarettes.^14^ Among Australian adolescents and adults aware of ONPs, 68% perceived them as harmful to health, while 38% perceived their harm as comparable to nicotine replacement therapy (NRT).^55^ In Quebec adolescents, those perceiving ONPs as low-risk were 10 times more likely to use them currently than those perceiving them as high-risk.^29^

#### Subjective ratings

Nine new randomized trials (three industry-funded, all BAT) examined product appeal and subjective ratings after ONP use.

##### Non-industry-funded studies

In a three-visit study, adult daily smokers were randomized to use wintergreen-flavored 3 mg ONPs containing either tobacco-derived or tobacco-free (synthetic) nicotine for 30 minutes. Both ONPs received moderate product liking scores and comparable withdrawal and craving relief. Those using tobacco-derived ONP reported more unfavorable sensory effects, such as tingling and burning sensations.^56^ Among adult daily SLT users assigned to two 30-minute ONP use sessions, 2 mg ONP use was associated with smaller reductions in cravings compared to 8 mg ONP and own-brand SLT use.^57^ In a single-visit study of young adult past 30-day e-cigarette and dual e-cigarette/cigarette users, 5-minute use of nicotine gum received higher appeal in terms of sensory attributes than 4 mg fruit or mint ONP use.^58^ In a three-visit study, adult daily moist snuff users rated both 6 mg ONPs with a lower vs. higher portion of freebase nicotine as moderately appealing, with comparable scores for withdrawal symptoms, craving relief, subjective effects, and aversion. Own-brand moist snuff received the highest scores for product appeal and craving relief.^59^

A five-arm study with adult daily smokers randomized to 20-minute use of either mint-flavored 6 mg, 20 mg, 30 mg ONPs, nicotine-free oral pouches, or cigarettes, found that all products, including nicotine-free pouches, reduced acute cigarette craving.^60^ In a 30-minute single-use study, exclusive daily Swedish snus users (≥ 8 mg/pouch) were randomized to each of five products (6 mg and 9 mg mint-flavored ONP, 11.2 mg spearmint-flavored ONP, 11.2 mg bergamot-flavored Swedish snus, 4 mg mint-flavored NRT nicotine gum) on separate days for 5 days. ONPs and snus received comparable scores for positive sensations (e.g., satisfaction, craving reduction) and negative sensations (e.g., aversion). NRT received the lowest scores for positive sensations and intention to use the product again, and the highest score for aversion. Both 9 mg and 11.2 mg ONPs and snus received comparable scores for intention to use again, while the 6 mg ONP scored lower.^61^

##### Industry-funded studies

In a series of in-clinic confinement studies, adult daily smokers were randomized to daily use of 5 different flavors of 4–12 mg ONPs for 30 minutes (followed by either NRT gum or cigarette use) for 6-8 days. Positive product effects, product liking, intentions to use again, and reduction in urges to smoke were consistently lower for ONPs than cigarettes, and comparable to the NRT gum. Higher nicotine strength ONPs showed greater reductions in urges to smoke, but elicited lower product liking and intent to use again, and higher negative product effects compared to lower nicotine strength ONPs, indicating less favorable subjective experiences.^62^ In another in-clinic confinement study, adult daily snus or ONP users were randomized to a single use of 11 mg or 20 mg ONPs for 10, 20, or 30 minutes daily for 7 days. All arms reported similar scores for product liking and intent to use again, while the 30-minute use of 20 mg ONP received the lowest scores.^63^ In adult daily smokers, product liking scores were neutral and comparable between 4 mg and 7 mg ONPs, regardless of flavors, after a single 45-minute daily use of one ONP during a 10-day confinement.^64^

#### ONP advertising

While the ONP market dynamics, sales data, and industry marketing strategies are reported later in this review, this section examines individual-level exposure to ONP marketing and its behavioral effects.

In the US, self-reported past exposure to tobacco product ads was positively associated with lifetime and current ONP use among adolescents.^19^ Exposure to ONP marketing, discounts, and coupons increased the risk of current ONP use among adults and was associated with ONP experimentation among adult current and former tobacco users.^24,50^

Brief exposure to an ONP ad featuring content appealing to youth was followed by more favorable ONP perceptions and greater intentions to use by young adults, particularly among prior tobacco users, females, and Black respondents.^51^ In a BAT-funded study, adult current tobacco experimenters reported the highest purchase intent and product appeal ratings after viewing an ONP ad, followed by current tobacco users and current non-tobacco users. Former and never-users of tobacco reported the lowest scores.^65^ In a tobacco-naïve population ages 15+ in the European Union, past exposure to ONP marketing was associated with higher product appeal, particularly in males and ages 15-24.^66^ In contrast, prior exposure to ONP ads was not associated with increased susceptibility to ONP use among Australian adolescents and young adults.^53^

### Product characteristics

#### Nicotine content and release from pouches

A US content analysis of 70 oral tobacco-free nicotine products found that ONPs contained the highest levels of total nicotine (5.71–31.5 mg/g), followed by loose leaf, toothpicks, lozenges, and gum. ONPs contained the highest total nicotine per product (1.41–8.11 mg/pouch) and highest freebase nicotine (2.15–16.8 mg/g). The proportion of freebase nicotine increased with increasing labeled nicotine strength and varied across ONP brands.^67^ Another content analysis of 50 ONPs showed that despite containing nearly 50% less nicotine than traditional tobacco pouches (6.4 ± 3.5 vs. 12.3 ± 8.2 mg/pouch), ONPs contained a higher portion of freebase nicotine than tobacco pouches (63.4% ± 25.2% vs. 47.2% ± 34.4%), resulting in a similar, but more rapid, nicotine release profile. Wide variations were observed across ONP brands and within different flavors and nicotine strengths of the same brands.^68^ Among 31 ONPs commercially available in Germany, the total measured nicotine ranged between 2.20 and 56 mg/pouch.^69^ The measured total nicotine levels in ONPs were generally the same or less than those reported on the label.^67,69^ In a BAT-funded study, the extraction of nicotine from ONPs increased with higher nicotine strength and longer duration of use, with the fastest rate of extraction within the first 10 minutes of use.^63^

#### Pharmacokinetics

Four of eight new RCTs on ONPs’ pharmacokinetic properties were industry-funded (BAT, Japan Tobacco International) (Supplementary Table S4).

##### Non-industry-funded studies

Among ONPs available in Germany, 30 mg ONP showed the highest overall (AUC) and maximum plasma nicotine concentration (C_max_) in adult daily smokers during a 4-hour assessment period, followed by cigarette, 20 mg ONP, and 6 mg ONP. Time to reach maximum concentration (T_max_) was shortest for the cigarette (5 minutes) and longest for 6 mg ONP (20 minutes).^60^ Compared to ONPs with synthetic nicotine, ONPs with tobacco-derived nicotine of the same strength and flavor (3 mg, wintergreen) showed a higher C_max_ in adult daily smokers at 30 minutes post-use.^56^ In adult daily SLT users, the mean nicotine concentration measured in plasma samples after 30-minute use sessions of 2 mg, 4 mg, and 8 mg ONPs increased in a nicotine content-dependent manner, with 8 mg ONPs delivering comparable or greater nicotine than SLT following the same use duration.^57^ Among adult daily moist snuff users, mean plasma nicotine concentration at 30 minutes of use, C_max_, and AUC were twice as high for the ONP with a higher vs. lower proportion of freebase nicotine of the same strength (6 mg), and comparable to moist snuff. T_max_ was comparable across products (36-42 minutes).^59^

##### Industry-funded-studies

Nicotine uptake from ONPs in adult daily smokers generally increased with higher nicotine strength and did not differ by flavor.^62, 64^ Compared to cigarettes, AUC within 15 minutes and 4 hours post-use, and C_max_ were lower from 4 mg and 8 mg ONPs, but comparable or higher from the 12 mg and 8 mg “mini” ONPs. The pharmacokinetic profile of the 4 mg “mini” was similar to that of 4 mg NRT gum. T_max_ was comparable across all ONPs and the NRT gum (35–40 minutes), and slower than that of a cigarette (5–7 minutes).^62^ Nicotine uptake (C_max_ and AUC within 4 hours post-use) from 11 mg and 20 mg ONPs in adult daily snus or ONP users increased with nicotine strength and duration of use. The C_max_ for the 20 mg ONP was higher than any previously reported values for ONPs and substantially greater than values reported for cigarettes.^63^ The trend for dose proportionality between nicotine strength of 6, 9, and 11.2 mg ONPs and nicotine uptake was observed among adult exclusive and daily Swedish snus users. C_max_ and AUC within 60 minutes post-use for all ONPs were higher than for 11.2 mg snus and 4 mg NRT gum, and twice as high for 11.2 mg ONP as for the 11.2 mg snus. ONPs delivered nicotine slightly faster than snus and NRT.^61^

### Toxicity

#### Chemical content

Four new studies provided evidence on the constituents of ONPs. An elemental analysis of six ONPs of various nicotine strengths and flavors confirmed the presence (not quantified) of aluminum, barium, calcium, chromium, copper, iron, manganese, sodium, scandium, strontium, and titanium.^70^ In ONPs of various flavors, high levels of a Xylitol sweetener (nearly 16 mg/pouch) and the total presence of 48 flavor compounds were reported.^71^ A content analysis of common flavorings used in tobacco products found that ONPs generally contained more flavorings than traditional tobacco pouches, with large variations across and within ONP brands. Menthol was most frequently present, even in fruit-flavored ONPs, especially in those with higher freebase nicotine.^68^ In a chemical analysis of ONPs of various flavors and nicotine strengths, all contained significant amounts of artificial sweeteners (0.3-1.2 mg/pouch), even those marketed as “unflavored.”^72^ In the VELO ONP brand, the sweetener content was higher in products with higher nicotine levels. Sweeteners at these levels reduced aversion, increased preference, and facilitated nicotine consumption in mice.^72^

#### In vitro toxicity

##### Non-industry-funded studies

An independent assessment of ONPs (10 mg, mint) found that 24-hour exposure of human monocyte leukemia cell lines to ONPs increased several pro-inflammatory cytokines beyond those observed with a pure nicotine solution control.^73^

##### Industry-funded studies

In a BAT-funded study, seven out of eight own-brand ONPs were deemed non-cytotoxic, non-mutagenic, and non-genotoxic after a 24-hour exposure in hamster ovary cells, while one (8 mg; spice flavor) was found cytotoxic and genotoxic at high nicotine equivalent concentrations exceeding those of a cigarette. The comparator ONP (ZYN, 6 mg, wintergreen) and snus (8 mg, mint) were found non-cytotoxic, non-mutagenic, and non-genotoxic, whereas cigarettes were positive in all three assays.^74^ An Altria-funded assessment of seven nicotine degradants and impurities in own-brand ONPs (6 mg, 9 mg, 12 mg, wintergreen, mint, tobacco) concluded that five did not exceed health risks of nicotine itself, while two exceeded levels of toxicity compared to pure nicotine.^75^

#### Human studies

Four new studies (one industry-funded) assessed biomarkers in humans. A study using PATH data found higher levels of nicotine metabolites in current exclusive ONP users than in exclusive vapers, and comparable to cigarette smokers, albeit based on a small sample size.^76^ Levels of minor tobacco alkaloids, cadmium, and uranium were lower in ONP users compared to smokers, and comparable to vapers. Lead levels were lower in ONP users compared to users of cigarettes and vapers, and comparable to no tobacco use.^76^ In a 4-week RCT, despite reducing smoking in adult daily smokers, dual use with ONPs did not reduce tobacco-specific nitrosamine levels.^47^ A 20-minute use of ONPs (6 mg, 20 mg, 30 mg) showed a dose-dependent increase in adult daily smokers’ heart rate, with the highest increase observed for 30 mg ONP and cigarette. The 20 mg and 30 mg ONPs elevated peripheral and central systolic blood pressure and arterial stiffness, comparable to cigarette.^60^ In an Altria-funded RCT, a reduction in gingivitis and enamel staining at 24 weeks was observed in adult current daily smokers who switched to ONPs compared to continued smoking.^17^

### Marketing and sales

#### Market share and sales data

An analysis of US Nielsen sales data (excluding online and specialty shops) found that ONP sales have more than tripled from 327 million units in July 2021 to 1046 million in May 2024, with sales revenue rising from US$61 million to US$232 million. ZYN, on!, Rogue, and VELO brands accounted for 99.8% of total sales, with ZYN leading the sales. Overall, sales were dominated by mint and wintergreen flavors (63%) and 3 mg and 6 mg strengths (67%), with faster growth in higher-strength (>4 mg) ONPs.^77^

#### Advertising expenditures and industry marketing strategies

From 2016 to 2023, Velo (BAT) ad expenditures in the US were mostly through TV (60%) and radio (30%), whereas ZYN (Swedish Match) spent most on online display and videos (79%). Of total ONP brand ad expenditures, Velo spent substantially more (89.8%, $26.3 million) than ZYN (5.7%, $1.7 million). Since 2022, ad expenditures for Velo dropped to $0 and ZYN became the leader in ad expenditures.^78^

A content analysis of Nordic Spirit Instagram ads between 2019 and 2023 identified four major themes that target young people, including “fun and freedom”, “Nordic happiness”, “fuss-free and discreet use”, and “healthy and scientifically rational”.^79^ Among Instagram posts promoting synthetic nicotine products in 2020–2022, most featured ONPs (52.9%), followed by e-cigarettes (30.6%) and flavored e-liquids (14.1%).^80^

From 2016 to 2022, US business-facing ONP ads focused on convenience (24%), retailer profitability (36%), product popularity and growing sales (24%), individual freedom (24%), and circumventing tobacco product restrictions (6%).^81^ A content analysis of major US ONP brand ads between 2021 and 2023 indicated a decline in marketing terms like “smoke-free”, “tobacco-free”, and “spit-free”, and an increase in claims of discrete and convenient use, and availability of coupons/discounts. Over 84% of ads were posted on brands’ social media accounts, with ZYN ads accounting for 55% of all platform ads.^82^ An analysis of US ZYN ads (excluding social media) identified digital media as the primary marketing channel between 2019 and 2023, with increased promotion of reward programs and “spit-free claims”, and decreased use of “tobacco-free” claims following PMI’s acquisition of ZYN in November 2022.^83^

#### Retail availability

In 2023, more than half of chain convenience stores and smoke/vape shops in three major US cities (San Francisco, New York City, and Philadelphia) sold ONPs. Greater availability of ONPs was observed in predominantly White and higher-income neighborhoods, and in neighborhoods with fewer youth residents.^84^ In Pakistan, nearly 8% of surveyed retailers in 2023 sold ONPs, with wider availability and variety in urban areas.^41,85^

## Discussion

Despite indications of growing market popularity of ONPs, the prevalence of current ONP use among the US adult general population remained below 1% through 2023. However, use is higher among those with a tobacco use history, consistent with findings from our previous review.^9^ Among US youth, nationally representative surveys indicate a slow but steady increase in ONP use prevalence, doubling between 2021 and 2024. Notably, the 2022/2023 prevalence of current youth ONP use appears similar to that of young adults (middle and high school students: 1-1.5% [NYTS, past 30-day use]; adults ages 18-24: 0.7% [TUS-CPS, defined as current use every day or some days]-1.5% [PATH, past 30-day use]), though direct comparisons are limited by survey heterogeneity. US sales of ONPs in this period have more than tripled. Disproportionately higher ONP use in some subpopulations, e.g., current tobacco and e-cigarette users, males, youth, and young adults, may be partially driving the increasing sales. Consistent with our previous review, awareness of ONPs in the US remains high, and is more common among youth and younger adults, and those with a tobacco use history.

Globally, higher ONP use prevalence, including among youth, was reported in countries both with a permissive regulatory approach, like Pakistan, and with restrictive ONP regulations, like Finland, Australia, and Canada. In the US and Pakistan, ONPs became the second most used product among youth after e-cigarettes, signaling their increasing popularity in younger segments.^20,21,44^ Further analysis of the impact of different regulatory regimes is warranted to inform future regulations.

The role of ONPs in the initiation and cessation of cigarettes and other nicotine delivery products remains unclear, lacking population-level longitudinal data on transition patterns.^86,87^ Consistent with our previous review, the present review of cross-sectional studies finds that current ONP use was more often associated with current cigarette and e-cigarette use than with former or never use. These findings might indicate supplemental use of ONPs among tobacco users, particularly in places where tobacco product use is prohibited. Indeed, ONPs are increasingly marketed as convenient and discrete products, and are commonly used by school-attending youth^20,21,29,39,40^ and employed adults.^26, 37^ Dual use may also reflect a transitional stage to a complete cessation of the initial tobacco product, but, to date, there is insufficient evidence on ONP as a smoking cessation aid. Both independent and industry-funded RCTs found that ONP use for up to 6 months, regardless of nicotine strength, significantly decreased cigarette and SLT consumption in adult daily smokers or SLT users, with minimal impact on smoking cessation. Independent, well-designed research on the longitudinal transitions of ONPs and other tobacco/nicotine products and randomized studies of the efficacy of ONP use in cigarette and e-cigarette cessation are needed to understand the substitutional and supplementary effects of ONPs. Future studies should consider multiple ONP use measures (e.g., everyday vs someday, frequency, intensity) to better distinguish transitions by extent of use.

Our earlier scoping review concluded that ONPs contained fewer harmful/potentially harmful constituents at lower levels than traditional tobacco products.^9^ Updated evidence suggests that ONPs also contain substantial amounts of flavorings and artificial sweeteners. We note substantial variations in ONP flavorings within and across brands, with some manufacturers increasing their content with higher nicotine strengths and freebase nicotine content, likely to reduce aversion and facilitate nicotine consumption. With flavored ONPs commonly used by both youth and adults, the role of flavors in ONP initiation and in the reduction or cessation of tobacco use remains unclear and merits further research. Further, collective findings from the present and earlier scoping reviews suggest that although ONPs are substantially less cytotoxic than cigarettes, ONP constituents beyond nicotine appear to induce inflammatory responses, oxidative stress, and cytotoxicity in short-term assays. Further independent research is needed to assess whether flavorings and sweeteners contribute to these cellular responses.

High levels of total and freebase nicotine in some ONPs raise further concerns. In some countries, nicotine content reaches 56 mg/pouch, while often not stated on the packaging.^69^ High proportions of freebase nicotine in some ONPs facilitate faster and greater nicotine absorption through buccal mucosa, potentially increasing nicotine dependence. Yet, ONPs with a high proportion of freebase nicotine (vs. low) do not appear to be superior in relieving cravings in SLT users,^56,59,60^ and their advantage in substitutability for cigarettes and other tobacco/nicotine products is unknown. In line with our previous review, new studies confirm that the nicotine uptake of some ONPs increases proportionally with nicotine dose, and the nicotine delivery of higher strength ONPs (> = 6 mg based on studies in our previous review and > =8 mg based on the updated review) often exceeds that of cigarettes in adult smokers. Notably, studies suggest significant variability in key ONP characteristics that could affect nicotine delivery from the product (including nicotine content, release rate, and proportion of freebase form). This variability suggests that various brands of ONPs may significantly differ in nicotine delivery and thus may have different abuse liability potential. Specifically, the assessment of abuse liability of ONPs should consider the speed of nicotine delivery (T_max_) since products with faster nicotine delivery are generally more addictive than products that deliver nicotine slowly. Due to the limited number of studies, a systematic assessment of all these factors is not currently feasible. Thus, a general conclusion about nicotine delivery applicable to the entire ONP product category cannot be made at this time.

While the pharmacokinetic properties of some ONPs may suggest a potential substitution for smoking, studies in our present and earlier review consistently found a lack of appeal compared to cigarettes, suggesting that smokers may seek behavioral and sensory attributes beyond nicotine delivery. Further, the delayed nicotine delivery time with ONPs compared to cigarettes may not provide immediate craving relief and lead to the use of multiple ONPs in a short time, also referred to as “stacking”.^88^ Such use, particularly of high-nicotine-strength ONPs, may result in an inadvertent overdose of nicotine and acute nicotine toxicity.^89^ Sales of high-nicotine strength ONPs have been on the rise,^77,90^ and are widely used by adolescents and young adults.^33,40^ The commonly reported lack of users’ knowledge about the ONP nicotine strength further contributes to this concern.^30,32,33^

The health impacts of ONP use remain largely understudied. Collectively, findings from our previous and present reviews indicate limited, mainly industry-funded evidence of short-term health benefits of switching to ONPs in adult smokers. Emerging evidence in the present and other reviews^91^ suggests that acute and regular use—especially at higher nicotine doses—may result in clinically relevant changes of specific markers of cardiovascular health, and may present elevated risk for vulnerable populations, such as individuals with cardiovascular disease.^69^ Similar effects were previously reported from the systemic nicotine absorption from smokeless tobacco.^92^ More research is needed to determine the short and long-term health effects of regular ONP use relative to traditional combustible and smokeless tobacco products, e-cigarettes, HTP, NRT, and no use.

US ONP advertising expenditures shifted dramatically between 2016 and 2023, as BAT (Velo) went from leading the market in ad expenditures to cutting its expenditures to $0 in 2022, and Swedish Match (ZYN) subsequently took the lead. This decline in Velo’s advertising may be due to the FDA’s March 2022 regulatory authority over non-tobacco nicotine products, and could represent a strategic pivot by BAT to divert marketing capital away from the US market, expanding into countries with lower regulatory barriers.^93^ Industry marketing strategies have also evolved in recent years, increasingly prioritizing social media to promote ONPs and emphasizing potentially youth-appealing claims about flavors, discreetness, and convenience. The industry widely employs celebrity endorsements, sponsorships of major sporting events, and targeted marketing to specific populations (e.g., athletes) to promote ONPs.^94–97^ These marketing tactics are reflected in the commonly reported reasons for ONP use identified in this review, with sport performance enhancement emerging as a new common reason. Furthermore, the industry appears to be moving away from the “tobacco-free” descriptor as a marketing tactic, possibly to mitigate complications with FDA authorization.^82,83^ Meanwhile, a heavily advertised variety of flavors and increasing promotion of coupons and reward programs may attract tobacco-naïve youth.^82,98^ Continuous monitoring of industry marketing strategies is essential, as exposure to ONP marketing was found to be associated with ONP use and product appeal among youth and adults. More research is needed on the effects of ONP marketing on tobacco-naïve populations and how these effects vary across countries with different marketing regulations.

Our review has several limitations. As a scoping review with broad inclusion criteria, we did not conduct a risk-of-bias assessment of the included studies or perform statistical analyses of the included data. Future work should focus on targeted systematic reviews with tighter scope restrictions, which are better suited to assessing the certainty and quality of the evidence. The associations with current ONP use should be interpreted with caution due to generally low sample sizes.

## Conclusion

ONPs may be a less hazardous alternative to traditional tobacco, but they are not risk-free. Co-use with other tobacco/nicotine products is high, and thus far, the effect of ONPs on smoking cessation remains unclear. Commercially available high-strength ONPs raise public health concerns and may increase nicotine dependence. Health effects of ONPs remain understudied. Future studies should evaluate their efficacy in long-term smoking cessation and their broader public health impact in tobacco-naïve populations.

## Supplementary Material

Supplementary Material

Supplementary material is available at *Nicotine & Tobacco Research* online.

## Figures and Tables

**Figure 1:**
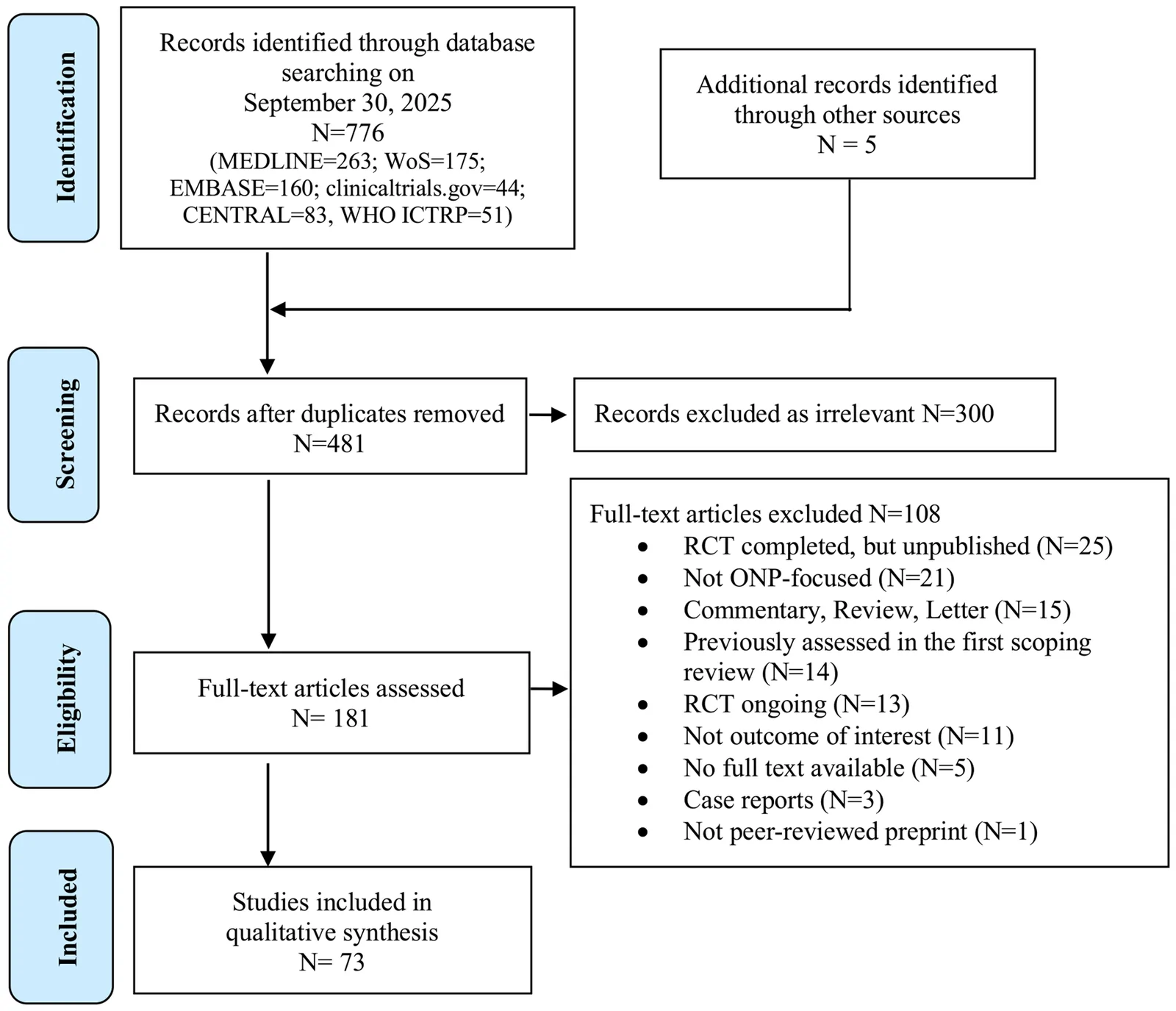
PRISMA flow-chart of eligibility screening.

## Data Availability

No new data were generated for this scoping review. All data analyzed are derived from previously published, publicly available studies identified through database searches. The full list of included studies is provided in Supplementary Table S1. The PubMed search strategy is provided in Supplementary File S1; search strategies for other databases are available from the corresponding author upon reasonable request.
