# Supplementary material for: Oral nicotine pouches and public health: evidence from an updated scoping review
Source: Nicotine Tob Res. Author manuscript; Available in PMC 2026 Oct 13. (PMC13660210; doi:10.1093/ntr/ntag203)
Supplement: Supplementary file 1 [file NIHMS2214286-supplement-Supplementary_Material.zip › Supplementary Table S1. Characteristics of all included studies R&R.docx]

**Oral Nicotine Pouches and Public Health: Evidence from an Updated Scoping Review**

Supplementary Table S1. Characteristics of the included studies from the first and updated reviews.

|  | **First author, year** | **Country** | **Funding** | **Outcome Category** | **Study Design** | **Sampling Strategy** | **Sample representativeness** | **Population** |
| --- | --- | --- | --- | --- | --- | --- | --- | --- |
| 1 | Keller-Hamilton et al., 2023^1^ | US, OH | Federal | Beliefs and perceptions; Product characteristics | Randomized trial | Non-probability (purposive) | Convenience Sample | Adult smokers ages ≥21 years (n=30) |
| 2 | Birdsey et al., 2023^2^ | US | Federal | Use patterns | Cross-sectional survey | Probability (stratified cluster) | Nationally representative | Middle and high school students (n=22,069) |
| 3 | Dowd et al., 2023^3^ | US | Federal | Use patterns | Cross-sectional survey | Non-probability | Convenience Sample | Adult current ONP users ages ≥18 years (n=118) |
| 4 | Long et al., 2023^4^ | US, OH | Federal | Beliefs and perceptions | Qualitative (focus groups) | Non-probability (purposive) | Convenience Sample | Adult smokers (n=19) and SLT users ages ≥21 years (n=18) |
| 5 | Morean et al., 2023^5^ | US | Federal | Beliefs and perceptions | Cross-sectional survey | Non-probability (quota/panel) | Convenience sample | Participants, ages 13+, oversampled current tobacco users (n=1,000) |
| 6 | Rinaldi et al., 2023^6^ | Germany | Federal | Toxicity | In-vitro | N.A. | N.A. | N.A. |
| 7 | Schneller et al., 2023^7^ | US | Federal | Use patterns | Cross-sectional survey | Probability (stratified) | Nationally-representative | Adolescents, ages 16-19 |
| 8 | Vogel et al., 2023^8^ | US | Federal | Beliefs and perceptions | Randomized control trial | Non-probability (purposive) | Convenience sample | Young adults past-month tobacco/nicotine users ages 21–34 years and no intention to quit (n=47) |
| 9 | Azzopardi et al., 2023^9^ | Sweden/  Denmark | BAT | Toxicity/Health effects | Cross-sectional clinical study | Non-probability (purposive) | Convenience sample | Adult exclusive users of ONPs, and current (≥10 cigarettes/day), former and never smokers ages 19-55 (n=195) |
| 10 | Back et al., 2023^10^ | Sweden/  Canada | Swedish Match | Toxicity | Chemical Analysis | N.A. | N.A. | N.A. |
| 11 | Couch et al., 2023^11^ | US | Federal | Use patterns | Cross-sectional survey | Non-probability | Convenience sample | Adolescents and young adult ENDS users ages 14-20 (n=2,253) |
| 12 | Gaiha et al., 2023^12^ | US | Federal | Use patterns | Cross-sectional survey | Non-probability (quota/panel) | Convenience sample | Adolescents, young adults, and adults ages 13–40 (n = 6,131) |
| 13 | Keller-Hamilton et al., 2023^13^ | US, OH | Federal | Beliefs and perceptions | Randomized trial | Non-probability (purposive) | Convenience sample | Young adult men ages ≥18 years (n=239) |
| 14 | Kramer et al., 2023^14^ | US | Federal | Use patterns; Beliefs and perceptions | Cross-sectional survey | Probability (stratified cluster) | Nationally representative | Middle and high school students (n = 20, 413) |
| 15 | Ling et al., 2023^15^ | US | Federal | Marketing and sales | Content analysis | N.A. | N.A. | N.A. |
| 16 | Mallock-Ohnesorg et al., 2023^16^ | Germany | Federal | Toxicity | Chemical Analysis | N.A. | N.A. | N.A. |
| 17 | Mays et al., 2023^17^ | US, OH | Federal | Beliefs and perceptions | Cross-sectional experimental | Non-probability (purposive) | Convenience sample | Adult cigarette smokers,  ST (chew, snuff, dip, and snus) users, and non-users of cigarettes or ST ages ≥ 21(n=301) |
| 18 | Morean et al., 2023^18^ | US | Federal | Use patterns; Beliefs and perceptions | Cross-sectional survey | Non-probability (quota/panel) | Convenience sample | Young adults ages 18–25, oversampled current tobacco users (n=609) |
| 19 | Patel et al., 2023^19^ | US | Truth Initiative | Use patterns | Cross-sectional survey | Non-probability (quota/panel) | Convenience sample | Youth and young adults ages 15-24 (n = 7,832) |
| 20 | Rensch et al., 2023^20^ | US | Altria | Toxicity/Health effects | Open-label, randomized, controlled, in-clinic, 5-parallel-group study | Non-probability (purposive) | Convenience sample | Adult smokers ages 21-65 (n = 144) |
| 21 | Sparrock et al., 2023^21^ | US | Federal | Use patterns; Beliefs and perceptions | Cross-sectional survey | Non-probability (quota/panel; sample-matching to mimic a probability sample) | Nationally representative | Adult current and former tobacco users ages ≥ 21 (n=1,583). |
| 22 | Tackett et al., 2023^22^ | US, Southern CA | Federal | Beliefs and perceptions | Cross-sectional survey | Non-probability (school-based) | Regional (Southern CA) | Adolescent (9^th^ and 10^th^ grade) never tobacco or nicotine users (n=1,289) |
| 23 | Tosakoon et al., 2023^23^ | US | Federal | Use patterns; Beliefs and perceptions | Cross-sectional survey | Non-probability (purposive; quota-based) | Convenience sample | Youth and young adults ages 18–34, oversampled cigarette/e-cigarette users (n=942). |
| 24 | Vogel et al., 2023^24^ | US, Southern CA | Federal | Beliefs and perceptions | Cross-sectional survey | Non-probability (school-based) | Regional (Southern CA) | Ninth- and tenth-grade student never tobacco users (n=3,129) |
| 25 | Emery et al, 2022^25^ | US | Federal | Marketing and Sales | Marketing surveillance data analysis | N.A. | N.A. | N.A. |
| 26 | Felicione et al., 2022^26^ | US | Federal | Use patterns; Beliefs and perceptions | Cross-sectional survey | Non-probability (quota/panel) | Convenience sample | Adult current and former smokers and e-cigarette users ages ≥18 (n=2,507) |
| 27 | Duan et al, 2022^27^ | US | Federal | Marketing and sales | Marketing surveillance data analysis | N.A. | N.A. | N.A. |
| 28 | Tattan-Birch et al., 2022^28^ | Great Britain (England, Scotland, and Wales) | Government | Use patterns | Cross-sectional survey | Probability (random) | Nationally representative | Adults ages ≥ 18 (n=25,698) |
| 29 | Harlow et al., 2022^29^ | US, Southern CA | Federal | Use patterns | Cross-sectional survey | Non-probability (school-based) | Regional (Southern CA) | 9^th^ and 10^th^ graders (n=3,516) |
| 30 | Morean et al., 2022^30^ | US | Federal | Use patterns; Beliefs and perceptions | Cross-sectional survey | Non-probability (quota/panel) | Convenience sample | Young adults ages  18-25, oversampled current e-cigarette and other tobacco product users (n=630) |
| 31 | Alizadehgharib et al., 2022^31^ | Sweden | Swedish Match | Toxicity/Health effects | Clinical and in-vitro study | Non-probability | Convenience sample | Healthy adult Swedish snus users ages ≥ 19 (n=60) |
| 32 | McEwan et al, 2022^32^ | Sweden | BAT | Product characteristics; Beliefs and perceptions | Randomized, controlled, crossover clinical study | Non-probability | Convenience sample | Healthy adult current dual users of snus and combustible cigarettes ages 19-55 (n=35) |
| 33 | Chapman et al., 2022^33^ | Sweden | Imperial Brands | Product characteristics; Beliefs and perceptions | Randomized, controlled open-label, cross-over clinical study | Non-probability | Convenience sample | Healthy adult users of traditional tobacco products  (cigarettes and Scandinavian snus) ages ≥ 19 (n=24) |
| 34 | Azzopardi et al., 2022^34^ | Canada | Imperial Tobacco Canada | Product characteristics; Beliefs and perceptions | Randomized crossover clinical study | Non-probability | Convenience sample | Healthy adult smokers ages 15-55 (n=34). |
| 35 | Vogel et al., 2022^35^ | US, Southern CA | Federal | Beliefs and perceptions | Cross-sectional survey | Non-probability (convenience) | Regional (Southern CA) | Young adult never users of ONPs ages 19-23 (n=1,167) |
| 36 | Liu et al., 2022^36^ | US | Altria | Product characteristics; Beliefs and perceptions | Randomized, partial single-blind, 7-way crossover design | Non-probability | Convenience sample | Adult dual cigarette and moist SLT users ages 21-65 (n=30) |
| 37 | Gentzke et al, 2022^37^ | US | Federal | Use patterns | Cross-sectional survey | Probability (stratified cluster) | Nationally representative | Middle and high school students (N=27.41 million) |
| 38 | Jablonski et al., 2022^38^ | US | Enthalpy Analytical | Toxicity | Chemical analysis | N.A. | N.A. | N.A. |
| 39 | Mallock et al., 2022^39^ | Germany | Federal | Product characteristics; Toxicity | Chemical analysis | N.A. | N.A. | N.A. |
| 40 | Shaikh et al., 2022^40^ | US | Federal | Toxicity | In-vitro study | N.A. | N.A. | N.A. |
| 41 | Shaikh et al., 2022^41^ | US | Federal | Toxicity | In-vitro study | N.A. | N.A. | N.A. |
| 42 | Rose et al., 2022^42^ | US (New Jersey, Kentucky, North Carolina, and New York) | Federal | Marketing and sales | Cross-sectional analysis | N.A. | N.A. | N.A. |
| 43 | Miller-Holt, 2022^43^ | Canada, US | Japan Tobacco Int. | Toxicity | In-vitro study | N.A. | N.A. | N.A. |
| 44 | Yu et al., 2022^44^ | US | Imperial Brands | Toxicity | In-vitro study | N.A. | N.A. | N.A. |
| 45 | Park-Lee et al., 2022^45^ | US | Federal | Use patterns | Cross-sectional survey | Probability (stratified cluster) | Nationally representative | Middle and high school  students (N=2.51 million) |
| 46 | Majmundar et al., 2022^46^ | US | Federal | Marketing and sales | Sales data analysis | N.A. | N.A. | N.A. |
| 47 | Azzopardi et al., 2021^47^ | N/A | BAT | Toxicity | Chemical Analysis | N.A. | N.A. | N.A. |
| 48 | Stanfill et al., 2021^48^ | US | Federal | Product characteristics | Chemical Analysis | N.A. | N.A. | N.A. |
| 49 | Delnevo et al., 2021^49^ | US | Federal | Marketing and sales | Sales data analysis | N.A. | N.A. | N.A. |
| 50 | East et al., 2021^50^ | Canada, England, US | Federal | Use patterns | Cross-sectional survey | Non-probability | Convenience Sample | Adolescents ages 16-19 (n=11,838 in the US) |
| 51 | Dalrymple et al., 2021^51^ | N/A | BAT | Toxicity/Health effects | In-vitro study | N.A. | N.A. | N.A. |
| 52 | Rensch et al., 2021^52^ | N/A | Altria | Product characteristics; Beliefs and perceptions | Single-blind, randomized, 7-way crossover study | Non-probability | Convenience Sample | Healthy adult smokers ages 21-65 (n=42). |
| 53 | Brose et al, 2021^53^ | UK | Government | Use patterns; Beliefs and perceptions | Cross-sectional survey | Non-probability (quota for age, gender and geographical region to mimic a probability sample) | Nationally representative | Adult current or former smokers and/or ENDS users ages ≥18 years (n=3,883) |
| 54 | East et al., 2021^54^ | N/A | BAT | Toxicity | In-vitro study | N.A. | N.A. | N.A. |
| 55 | Marynak et al, 2021^55^ | US | Federal | Marketing and Sales | Sales data analysis | N.A. | N.A. | N.A. |
| 56 | Havermans et al., 2021^56^ | Netherlands | Government | Use patterns; Beliefs and perceptions | Cross-sectional survey | Probability (stratified) | Nationally representative | Youth and adults ages 13 and over (n=5805) |
| 57 | Li et al., 2021^57^ | Australia, Canada, England, US | Government | Use patterns | Cross-sectional survey | Non-probability | Convenience Sample | Adult current cigarette smokers (n=9,112) and recent ex-smokers (n=1,184) ages ≥18 years |
| 58 | Hrywna et al., 2021^58^ | US | Federal | Use patterns; Beliefs and perceptions | Cross-sectional survey | Probability | Nationally representative | Current adult smokers ages ≥18 years (n=1,018) |
| 59 | Czaplicki et al, 2021^59^ | US | Truth Initiative | Marketing and sales | Marketing surveillance data analysis | N.A. | N.A. | N.A. |
| 60 | Lunell et al., 2020^60^ | Sweden | Swedish Match | Product characteristics | An open, randomized, crossover, single-dose administration trial | Non-probability | Convenience sample | Adult snus users >19 years (n=29) |
| 61 | Plurphanswat et al., 2020^61^ | US | Tobacco Industry | Use patterns; Beliefs and perceptions. | Cross-sectional survey | Non-probability | Convenience sample | Adult current ZYN users ages ≥18 (n=1,266) |
| 62 | Bishop et al., 2020^62^ | N/A | BAT | Toxicity | In-vitro study | N.A. | N.A. | N.A. |
| **Newly added studies** | | | | | | | | |
| 63 | Akhlague et al., 2024^63^ | Pakistan | Independent | Use patterns; Beliefs and perceptions; Marketing and sales | Mix-method | Non-probability (purposive) | Convenience sample | Adult ≥18 current ONP users (n=90; n=12). ONP retailers (n=90). |
| 64 | Avila et al., 2024^64^ | US (RI) | Academic | Use patterns | Pilot randomized control trial | Non-probability (purposive) | Convenience sample | Adult smokers with low SES ages ≥ 21+ (n=33) |
| 65 | Becker et al., 2023^65^ | US | Altria | Use patterns | Multi-site, observational | Non-probability (quota/panel) | Convenience sample | Adult smokers and /or SLT users ages 18-64 with no intention to quit (n=1,147) |
| 66 | Brierley et al., 2025^66^ | Australia | Government | Beliefs and perceptions | Cross-sectional survey | Non-probability (quota/panel) | Convenience sample | Adolescents and younger adults ages 16–39 (n=817) |
| 67 | Brose et al., 2025^67^ | UK | Government | Use patterns | Cross-sectional survey | Probability (stratified) | Nationally representative | Youth ages 11-18 (n=2584). Adults ≥ 18 (n=12,247 -13,266) |
| 68 | Campbell et al., 2022^68^ | US | BAT | Use patterns | Pilot multisite, open-label, 8-week, prospective observational study | Non-probability | Convenience sample | Young adult daily cigarette smokers ages ≥ 21 (n=100) |
| 69 | Clark et al., 2024^69^ | US (NC) | Federal | Beliefs and perceptions | Qualitative (interviews) | Non-probability (purposive) | Convenience sample | Adult ≥ 18 current smokers (n=30) |
| 70 | Czaplicki et al., 2024^70^ | US | Federal | Marketing and sales | Ad content analysis | N.A. | N.A. | N.A. |
| 71 | Dai & Leventhal 2024^71^ | US | Federal | Use patterns | Cross-sectional survey | Probability (stratified) | Nationally representative | Adults ages ≥ 18 (n=39,558) |
| 72 | Freeland et al., 2023^72^ | US | BAT | Beliefs and perceptions | Cross-sectional survey | Non-probability (quota/panel) | Convenience sample | Adults ages ≥ 18 (n=about 49,000) |
| 73 | Giovenco et al., 2025^73^ | US (New York City, San Francisco, Philadelphia) | Federal | Marketing and sales | Cross-sectional survey | N.A. | N.A. | N.A. |
| 74 | Hanewinkel & Hansen, 2024^74^ | Germany | Federal | Use patterns | Cross-sectional survey | Non-probability (school-based cluster) | Convenience sample | School students ages 9-17 (n=12,655) |
| 75 | Jabba et al., 2024^75^ | US | Federal | Product characteristics | Chemical analysis/In-vivo | N.A. | N.A. | N.A. |
| 76 | Jackson et al., 2024^76^ | England | Government | Use patterns | Cross-sectional survey | Probability (stratified) | Nationally representative | Adults ages ≥ 18 (n=197,266) |
| 77 | Jackson et al., 2025^77^ | England | Government | Use patterns | Cross-sectional survey | Probability (stratified) | Nationally representative | Adults ages ≥16 past-year daily or occasional smokers trying to quit (n=25,094) |
| 78 | Jamal et al., 2024^78^ | US | Federal | Use patterns | Cross-sectional survey | Probability (stratified cluster) | Nationally representative | Middle and high school students, (n=29,861) |
| 79 | Jankowski & Rees, 2024^79^ | Poland | None | Use patterns; Beliefs and perceptions | Cross-sectional survey | Non-probability (quota/stratification applied to mimic a probability sample) | Nationally representative | Adults ages ≥ 18 (n=1,080) |
| 80 | Jongenelis et al., 2024 ^80^ | Australia | Government | Use patterns; Beliefs and perceptions | Cross-sectional survey | Non-probability (purposive) | Convenience sample | Youth and adults ages 16–39 years (n=1,598) |
| 81 | Kanobe et al., 2025^81^ | US | BAT | Beliefs and perceptions; Product characteristics | Randomized, open-label, crossover, in-clinic confinement studies | Non-probability (purposive) | Convenience sample | Adults ages ≥ 21 daily smokers (study 1 n=41; study 2 n=43; study 3 n=36) |
| 82 | Keller-Hamilton et al., 2024^82^ | US (OH) | Federal | Beliefs and perceptions; Product characteristics | Three-visit single-blind, randomized crossover  study | Non-probability | Convenience sample | Adult current smokers ages ≥ 21 (n=18) |
| 83 | Keyser et al., 2024^83^ | US | BAT | Toxicity | In-vitro | N.A. | N.A. | N.A. |
| 84 | Liu et al., 2025^84^ | N/A | Altria | Use patterns; Health effects | Randomized, open-label, parallel-group study | Non-probability | Convenience sample | Adult current smokers ages 23–65 (n=100) |
| 85 | Liu & Halpern-Felsher 2024^85^ | US | Federal | Use patterns | Cross-sectional survey | Non-probability (quota/panel) | Convenience sample | Adolescents (ages 13- 20), young adults (ages 21–24), and adults (ages 25–40) (n = 6,131) |
| 86 | Liu & McCauley et al., 2024^86^ | US | Federal | Beliefs and perceptions | Cross-sectional survey | Non-probability (quota/panel) | Convenience sample | Adolescents (ages 13- 20), young adults (ages 21–24), and adults (ages 25–40) (n = 6,131) |
| 87 | Mallock-Ohnesorg et al., 2024 ^87^ | Germany | Federal | Beliefs and perceptions; Product characteristics; Health effects | Single-center, five-arm, crossover study | Non-probability (purposive) | Convenience sample | Adults ages 18-55 daily smokers (n=15) |
| 88 | McEwan et al., 2023^88^ | US | BAT | Beliefs and perceptions; Product characteristics | Randomized, open-labelled, controlled, crossover clinical study | Non-probability (purposive) | Convenience sample | Adult daily smokers  (n=42) |
| 89 | Mohr et al., 2024^89^ | Germany | Academic | Toxicity | In-vitro | N.A. | N.A. | N.A. |
| 90 | Munawar et al., 2025^90^ | Pakistan | Government | Toxicity | Chemical analysis | N.A. | N.A. | N.A. |
| 91 | Nawaz et al., 2023^91^ | Pakistan | None | Use patterns; Beliefs and perceptions | Cross-sectional survey | Non-probability | Convenience sample | Youth and adults ages 13-56 (n=1,749) |
| 92 | O‘Hagan et al., 2024^92^ | UK | None | Marketing and sales | Qualitative content analysis | N.A. | N.A. | N.A. |
| 93 | Ozga et al., 2024^93^ | US | Federal | Marketing and sales | Cross-sectional | N.A. | N.A. | N.A. |
| 94 | Park-Lee et al., 2024^94^ | US | Federal | Use patterns | Cross-sectional survey | Probability (stratified cluster) | Nationally representative | Students in middle and high school (n=29,861) |
| 95 | Peraza et al., 2024^95^ | US | Federal | Beliefs and perceptions | Single-blind, single-visit randomized study | Non-probability (purposive) | Convenience sample | Young adults ages 21-29 current e-cigarette and/  or dual e-cigarette and cigarette users (n =46) |
| 96 | Phan et al., 2024^96^ | US | Federal | Use patterns; Beliefs and perceptions | Cross-sectional survey | Non-probability (sample-matching and weighing applied to mimic a probability sample) | Nationally representative | Adult current and former tobacco users (≥21 years), with oversampling of Asian/Asian American and Black/African American (n =1,700) |
| 97 | Prasad & Edward 2025^97^ | Sweden/Denmark | BAT | Use patterns | Cross-sectional survey | Probability (random selection; quota) | Nationally representative | Adult ages 18–64 in Sweden (n = 7,003) and Denmark (n = 4,402) |
| 98 | Prasad et al., 2022^98^ | Sweden, Denmark, Germany and Switzerland | BAT | Use patterns; Beliefs and perceptions | Cross-sectional survey | Non-probability (purposive) | Convenience sample | Adult current ONP users ages 18-64 (n=550) |
| 99 | Reimann et al., 2024^99^ | Germany | Federal | Toxicity/Health effects | Chemical analysis | N.A. | N.A. | N.A. |
| 100 | Renard et al., 2024^100^ | Sweden | Japan Tobacco Int. | Beliefs and perceptions; Product characteristics | Randomized, open-label, ten-sequence, single-use, cross-over clinical study | Non-probability | Convenience sample | Adult Swedish snus users ages 19–64 years (n=30) |
| 101 | Reyes-Guzman et al., 2025^101^ | US | Federal | Use patterns | Cross-sectional survey | Probability (stratified) | Nationally representative | Adults ≥ 18 years (n=40,000) |
| 102 | Ross et al., 2024^102^ | US | Federal | Use patterns | Cross-sectional survey | Non-probability (quota/panel) | Convenience sample | Youth ages 15-20 who reported using or susceptibility  for use of little filtered cigars or cigarillos (n=680) |
| 103 | Ruokolainen et al., 2024^103^ | Finland | None | Use patterns | Cross-sectional survey | Probability (census-based) | Nationally representative | Youth ages 14-20 (n=151,224) |
| 104 | Shah et al., 2024^104^ | US | Academic | Marketing and sales | Content analysis | N.A. | N.A. | N.A. |
| 105 | Sharma et al., 2024^105^ | US | Federal | Marketing and sales | Content analysis | N.A. | N.A. | N.A. |
| 106 | Silver et al., 2024^106^ | US | None | Beliefs and perceptions | Experimental | Non-probability (quota/panel) | Convenience sample | Young adults ages 18-24, oversampled current cigarette users (n=1,450) |
| 107 | Tran et al., 2024^107^ | US | None | Product characteristics | Chemical analysis | N.A. | N.A. | N.A. |
| 108 | Watts et al, 2024^108^ | Australia | Government | Beliefs and perceptions | Qualitative | Non-probability | Convenience sample | Adolescents (n=98, ages 14-17) and young adults (n=112, ages 18-24) who vaped or had tried vaping in the past. |
| 109 | Aldeek et al, 2025^109^ | N/A | Altria | Product characteristics | Chemical analysis | N.A. | N.A. | N.A. |
| 110 | Alkharaan et al, 2025^110^ | Saudi Arabia | Academic | Use patterns; Beliefs and perceptions | Cross-sectional survey | Non-probability | Convenience sample | Adults ages ≥ 18 years (n=831) |
| 111 | Alwafi et al, 2025^111^ | Saudi Arabia | Academic | Use patterns | Cross-sectional survey | Non-probability | Convenience sample | Adult tobacco users ages ≥ 18 years (n=657) |
| 112 | Azzopardi et al, 2025^112^ | Sweden | BAT | Beliefs and perceptions; Product characteristics | Single-use open-label randomized seven-way  crossover study | Non-probability (purposive) | Convenience sample | Adults ages ≥ 18 daily snus and ONP users (n=35) |
| 113 | Battista et al., 2025^113^ | Canada, Quebec | Government | Use patterns; Beliefs and perceptions | Cross-sectional survey | Probability (school-based stratified) | Regionally representative (Quebec) | Students grades 7-11 (n=13,914) |
| 114 | Brierley et al, 2025^114^ | Australia | Government | Beliefs and perceptions | Cross-sectional survey |  |  | Adolescents, young adults, and adults ages 16-39 who reported being aware of ONPs (n =1,230) |
| 115 | Cohen et al, 2025^115^ | Pakistan | Bloomberg Initiative | Product characteristics | Chemical analysis | N.A. | N.A. | N.A. |
| 116 | Dai & Young, 2025^116^ | US | Federal | Toxicity/Health effects | Cross-sectional survey | Non-probability (quota/panel) | Convenience sample | Adults (n=4,527) |
| 117 | Delnevo et al, 2025^117^ | US | Federal | Use patterns | Cross-sectional survey | Probability (stratified) | Nationally representative | Adults ages ≥ 18 (n=110, 084) |
| 118 | Elsokkary et al, 2025^118^ | Saudi Arabia | Academic | Use patterns | Cross-sectional survey | Probability (stratified) | Nationally representative | Adults ages ≥ 18 (n=385) |
| 119 | Eversole et al, 2025^119^ | US | Federal | Product characteristics | Randomized controlled trial | Non-probability | Convenience sample | Daily adult SLT users (n=23) |
| 120 | Farzeen et al, 2025^120^ | Pakistan | Government | Use patterns; Beliefs and perceptions | Cross-sectional survey | Non-probability (stratified random sampling strategy used to ensure representativeness) | Nationally representative | 10–16-year-old children in school and out of school (n=14,232) |
| 121 | Farzeen et al, 2025^121^ | Pakistan | Government | Marketing and sales | Observational | N.A. | N.A. | N.A. |
| 122 | Feizy et al., 2025^122^ | US | Federal | Use patterns; Beliefs and perceptions | Cross-sectional survey | Probability (stratified cluster) | Nationally representative | Middle and high-school students (n=26,860) |
| 123 | Fucito et al., 2025^123^ | US | Federal | Use patterns; Toxicity | Open-label,  randomized pilot trial | Non-probability (purposive) | Convenience sample | Adult daily smokers ages ≥21 not wanting to quit (n=30) |
| 124 | Han et al, 2025^124^ | US | Federal | Use patterns | Cross-sectional survey | Probability (stratified cluster) | Nationally representative | 10-12 graders (n=10,146) |
| 125 | Harlow et al, 2025^125^ | US, South CA | Federal | Use patterns | Longitudinal survey | Non-probability (school-based) | Regional (Southern CA) | 9th graders past 6-month e-cigarette users (n=703) |
| 126 | He et al., 2025^126^ | US | Federal | Marketing and sales | Cross-sectional | N.A. | N.A. | N.A. |
| 127 | Keller-Hamilton et al., 2025^127^ | US, OH | Federal | Beliefs and perceptions; Product characteristics | Single-blind, randomized crossover human laboratory study | Non-probability (purposive) | Convenience sample | Adult ages ≥21 daily moist snuff users (n=62) |
| 128 | Lee et al, 2025^128^ | US | None | Use patterns; Beliefs and perceptions | Cross-sectional survey | Probability (stratified) | Nationally representative | Adults ages ≥18 (n = 27,712) |
| 129 | Lyu et al, 2025^129^ | US | Federal | Marketing and sales | Content analysis | N.A. | N.A. | N.A. |
| 130 | Page et al., 2025^130^ | US | Federal | Product characteristics | Chemical analysis | N.A. | N.A. | N.A. |
| 131 | Palmer et al., 2025^131^ | US | Federal | Use patterns | Cross-sectional survey | Probability (stratified) | Nationally representative | Youth ages 12-17 (n=10, 632) and adult ages ≥18 (n=29,754) |
| 132 | Taylor et al., 2025^132^ | UK | Government | Use patterns; Beliefs and perceptions | Experimental | Non-probability (quota/panel) | Convenience sample | Adults ages ≥18 (n=2,967) |
| 133 | Turner et al., 2025^133^ | Canada | Academic | Use patterns | Cross-sectional survey | Non-probability | Convenience sample | University or college students ages ≥ 18 (n=452) |
| 134 | Xin Li et al, 2025^134^ | European Union | Government | Beliefs and perceptions | Cross-sectional survey | Probability (stratified) | Multi-country representative | Tobacco-naïve population ages ≥15 (n=13,436) |
| 135 | Ruokolainen et al, 2025^135^ | Finland | None | Use patterns | Cross-sectional survey | Probability (stratified) | Nationally representative | Adolescents and adults ages 15–69 (n=3,857), with younger age groups (15–39 years) oversampled |

SLT= Smokeless Tobacco. ENDS= Electronic Nicotine Delivery System. SES= Socioeconomic Status. BAT=British American Tobacco. N/A=not available; n.a.= not applicable.

**References:**

1.	Keller-Hamilton B, Alalwan MA, Curran H, et al. Evaluating the effects of nicotine concentration on the appeal and nicotine delivery of oral nicotine pouches among rural and Appalachian adults who smoke cigarettes: A randomized cross-over study. *Addiction.* 2023.

2.	Birdsey J, Cornelius M, Jamal A, et al. Tobacco Product Use Among U.S. Middle and High School Students - National Youth Tobacco Survey, 2023. *MMWR Morb Mortal Wkly Rep.* 2023;72(44):1173-1182.

3.	Dowd AN, Thrul J, Czaplicki L, Kennedy RD, Moran MB, Spindle TR. A Cross-Sectional Survey on Oral Nicotine Pouches: Characterizing Use-Motives, Topography, Dependence Levels, and Adverse Events. *Nicotine Tob Res.* 2024;26(2):245-249.

4.	Long L, Alalwan MA, Keller-Hamilton B, et al. Perceptions of oral nicotine pouches & their marketing among Ohio Appalachia smokers and smokeless tobacco users. *PLoS ONE.* 2023;18(10):e0293597.

5.	Morean ME, Gueorguieva R, O'Malley S, Krishnan-Sarin S. Including the term 'tobacco-free nicotine' in the nicotine addiction warning label mandated by the US Food and Drug Administration alters risk perceptions and use intentions. *Tob Control.* 2023.

6.	Rinaldi S, Pieper E, Schulz T, et al. Oral nicotine pouches with an aftertaste? Part 2: in vitro toxicity in human gingival fibroblasts. *Arch Toxicol.* 2023;97(9):2343-2356.

7.	Schneller LM, Felicione NJ, Hammond D, Goniewicz ML, O'Connor RJ. Tobacco-Free Oral Nicotine Product Use Among Youth in the U.S., 2019-2021. *AJPM Focus.* 2023;2(1):100061.

8.	Vogel EA, Tackett AP, Unger JB, et al. Effects of flavour and modified risk claims on nicotine pouch perceptions and use intentions among young adults who use inhalable nicotine and tobacco products: a randomised controlled trial. *Tob Control.* 2023.

9.	Azzopardi D, Haswell LE, Frosina J, et al. Assessment of biomarkers of exposure and potential harm, and physiological and subjective health measures in exclusive users of nicotine pouches and current, former and never smokers. *Biomarkers.* 2023;28(1):118-129.

10.	Back S, Masser AE, Rutqvist LE, Lindholm J. Harmful and potentially harmful constituents (HPHCs) in two novel nicotine pouch products in comparison with regular smokeless tobacco products and pharmaceutical nicotine replacement therapy products (NRTs). *BMC Chem.* 2023;17(1):9.

11.	Couch ET, Halpern-Felsher B, Werts M, Chaffee BW. Use of Emerging and Conventional Oral Tobacco Among Adolescent and Young Adult E-Cigarette Users. *Subst Use Misuse.* 2023;58(2):283-288.

12.	Gaiha SM, Lin C, Lempert LK, Halpern-Felsher B. Use, marketing, and appeal of oral nicotine products among adolescents, young adults, and adults. *Addict Behav.* 2023;140:107632.

13.	Keller-Hamilton B, Curran H, Stevens EM, Zettler PJ, Mays D, Ferketich AK. Effects of "Tobacco Free" Language in Warning Labels on Perceptions of Electronic Cigarettes and Nicotine Pouches among Young Adult Men: A Randomized Trial. *Subst Use Misuse.* 2023;58(10):1302-1306.

14.	Kramer RD, Park-Lee E, Marynak KL, Jones JT, Sawdey MD, Cullen KA. Nicotine Pouch Awareness and Use Among Youth, National Youth Tobacco Survey, 2021. *Nicotine Tob Res.* 2023.

15.	Ling PM, Hrywna M, Talbot EM, Lewis MJ. Tobacco-Derived Nicotine Pouch Brands and Marketing Messages on Internet and Traditional Media: Content Analysis. *JMIR Form Res.* 2023;7:e39146.

16.	Mallock-Ohnesorg N, Rinaldi S, Malke S, et al. Oral nicotine pouches with an aftertaste? Part 1: screening and initial toxicological assessment of flavorings and other ingredients. *Arch Toxicol.* 2023.

17.	Mays D, Long L, Alalwan MA, et al. The Effects of Oral Nicotine Pouch Packaging Features on Adult Tobacco Users' and Non-Users' Product Perceptions. *Int J Environ Res Public Health.* 2023;20(4).

18.	Morean ME, Bold KW, Davis DR, Kong G, Krishnan-Sarin S, Camenga DR. Awareness, susceptibility, and use of oral nicotine pouches and comparative risk perceptions with smokeless tobacco among young adults in the United States. *PLoS ONE.* 2023;18(1):e0281235.

19.	Patel M, Kierstead EC, Kreslake J, Schillo BA. Patterns of oral nicotine pouch use among U.S. adolescents and young adults. *Prev Med Rep.* 2023;34:102239.

20.	Rensch J, Edmiston J, Wang J, Jin X, Sarkar M. A Randomized, Controlled Study to Assess Changes in Biomarkers of Exposures Among Adults Who Smoke That Switch to Oral Nicotine Pouch Products Relative to Continuing Smoking or Stopping All Tobacco Use. *J Clin Pharmacol.* 2023.

21.	Sparrock LS, Phan L, Chen-Sankey J, et al. Nicotine Pouch: Awareness, Beliefs, Use, and Susceptibility among Current Tobacco Users in the United States, 2021. *Int J Environ Res Public Health.* 2023;20(3).

22.	Tackett AP, Wong M, Cho J, et al. Willingness to Use Commercial Nicotine Gums, Lozenges, and Gummies Among Nontobacco Using Adolescents in Southern California. *J Adolesc Health.* 2023;72(2):277-286.

23.	Tosakoon S, Romm KF, Berg CJ. Nicotine pouch awareness, use and perceptions among young adults from six metropolitan statistical areas in the United States. *Tob Prev Cessat.* 2023;9:19.

24.	Vogel EA, Barrington-Trimis JL, Harlow AF, et al. Prevalence of and disparities in adolescents' susceptibility to novel oral nicotine products marketed as "tobacco-free". *Prev Med.* 2023;166:107387.

25.	Emery SL, Binns S, Carter CC, Rose SW, Kostygina G. Characterising advertising strategies and expenditures for conventional and newer smokeless tobacco products. *Tob Control.* 2022.

26.	Felicione NJ, Schneller LM, Goniewicz ML, et al. Oral Nicotine Product Awareness and Use Among People Who Smoke and Vape in the U.S. *Am J Prev Med.* 2022;63(4):611-618.

27.	Duan Z, Henriksen L, Vallone D, et al. Nicotine pouch marketing strategies in the USA: an analysis of Zyn, On! and Velo. *Tob Control.* 2022.

28.	Tattan-Birch H, Jackson SE, Dockrell M, Brown J. Tobacco-free Nicotine Pouch Use in Great Britain: A Representative Population Survey 2020-2021. *Nicotine Tob Res.* 2022;24(9):1509-1512.

29.	Harlow AF, Vogel EA, Tackett AP, et al. Adolescent Use of Flavored Non-Tobacco Oral Nicotine Products. *Pediatrics.* 2022;150(3).

30.	Morean ME, Bold KW, Davis DR, Kong G, Krishnan-Sarin S, Camenga DR. "Tobacco-free" Nicotine Pouches: Risk Perceptions, Awareness, Susceptibility, and Use among Young Adults in the United States. *Nicotine Tob Res.* 2022.

31.	Alizadehgharib S, Lehrkinder A, Alshabeeb A, Östberg AK, Lingström P. The effect of a non-tobacco-based nicotine pouch on mucosal lesions caused by Swedish smokeless tobacco (snus). *Eur J Oral Sci.* 2022;130(4):e12885.

32.	McEwan M, Azzopardi D, Gale N, et al. A Randomised Study to Investigate the Nicotine Pharmacokinetics of Oral Nicotine Pouches and a Combustible Cigarette. *Eur J Drug Metab Pharmacokinet.* 2022;47(2):211-221.

33.	Chapman F, McDermott S, Rudd K, et al. A randomised, open-label, cross-over clinical study to evaluate the pharmacokinetic, pharmacodynamic and safety and tolerability profiles of tobacco-free oral nicotine pouches relative to cigarettes. *Psychopharmacology (Berl).* 2022;239(9):2931-2943.

34.	Azzopardi D, Ebajemito J, McEwan M, et al. A randomised study to assess the nicotine pharmacokinetics of an oral nicotine pouch and two nicotine replacement therapy products. *Sci Rep.* 2022;12(1):6949.

35.	Vogel EA, Barrington-Trimis JL, Kechter A, et al. Differences in Young Adults' Perceptions of and Willingness to Use Nicotine Pouches by Tobacco Use Status. *IJERPH.* 2022;19(5).

36.	Liu J, Rensch J, Wang J, et al. Nicotine pharmacokinetics and subjective responses after using nicotine pouches with different nicotine levels compared to combustible cigarettes and moist smokeless tobacco in adult tobacco users. *Psychopharmacology (Berl).* 2022;239(9):2863-2873.

37.	Gentzke AS, Wang TW, Cornelius M, et al. Tobacco Product Use and Associated Factors Among Middle and High School Students - National Youth Tobacco Survey, United States, 2021. *MMWR Surveill Summ.* 2022;71(5):1-29.

38.	Jablonski JJ, Cheetham AG, Martin AM. Market Survey of Modern Oral Nicotine Products: Determination of Select HPHCs and Comparison to Traditional Smokeless Tobacco Products. *SEPARATIONS.* 2022;9(3).

39.	Mallock N, Schulz T, Malke S, Dreiack N, Laux P, Luch A. Levels of nicotine and tobacco-specific nitrosamines in oral nicotine pouches. *Tob Control.*

40.	Shaikh S, Tung WC, Lucas J, Yogeswaran S, Li D, Rahman I. Flavor Classification/Categorization and Differential Toxicity of Oral Nicotine Pouches (ONPs) in Lung Epithelial Cells. *bioRxiv.* 2022.

41.	Shaikh SB, Tung WC, Pang C, Lucas J, Li D, Rahman I. Flavor Classification/Categorization and Differential Toxicity of Oral Nicotine Pouches (ONPs) in Oral Gingival Epithelial Cells and Bronchial Epithelial Cells. *Toxics.* 2022;10(11).

42.	Rose SW, Annabathula A, Westneat S, et al. Neighborhood distribution of availability of newer tobacco products: A four-site study, 2021. *medRxiv.* 2022.

43.	Miller-Holt J, Baskerville-Abraham I, Sakimura M, Fukushima T, Puglisi A, Gafner J. In vitro evaluation of mutagenic, cytotoxic, genotoxic and oral irritation potential of nicotine pouch products. *Toxicology Reports.* 2022;9:1316-1324.

44.	Yu F, Rudd K, Pour SJ, et al. Preclinical Assessment of Tobacco-Free Nicotine Pouches Demonstrates Reduced in Vitro Toxicity Compared with Tobacco Snus and Combustible Cigarette Smoke. *Applied In Vitro Toxicology.* 2022;8(1):24-35.

45.	Park-Lee E, Ren C, Cooper M, Cornelius M, Jamal A, Cullen KA. Tobacco Product Use Among Middle and High School Students - United States, 2022. *MMWR Morb Mortal Wkly Rep.* 2022;71(45):1429-1435.

46.	Majmundar A, Okitondo C, Xue A, Asare S, Bandi P, Nargis N. Nicotine Pouch Sales Trends in the US by Volume and Nicotine Concentration Levels From 2019 to 2022. *JAMA Netw Open.* 2022;5(11):e2242235.

47.	Azzopardi D, Liu C, Murphy J. Chemical characterization of tobacco-free "modern" oral nicotine pouches and their position on the toxicant and risk continuums. *Drug Chem Toxicol.* 2022;45(5):2246-2254.

48.	Stanfill S, Tran H, Tyx R, et al. Characterization of Total and Unprotonated (Free) Nicotine Content of Nicotine Pouch Products. *Nicotine Tob Res.* 2021;23(9):1590-1596.

49.	Delnevo CD, Hrywna M, Lo EJM, Wackowski OA. Examining Market Trends in Smokeless Tobacco Sales in the United States: 2011-2019. *NICOTINE & TOBACCO RESEARCH.* 2021;23(8):1420-1424.

50.	East KA, Reid JL, Rynard VL, Hammond D. Trends and Patterns of Tobacco and Nicotine Product Use Among Youth in Canada, England, and the United States From 2017 to 2019. *J Adolesc Health.* 2021;69(3):447-456.

51.	Dalrymple A, Bean EJ, Badrock TC, et al. Enamel staining with e-cigarettes, tobacco heating products and modern oral nicotine products compared with cigarettes and snus: An in vitro study. *Am J Dent.* 2021;34(1):3-9.

52.	Rensch J, Liu J, Wang J, Vansickel A, Edmiston J, Sarkar M. Nicotine pharmacokinetics and subjective response among adult smokers using different flavors of on!® nicotine pouches compared to combustible cigarettes. *Psychopharmacology (Berl).* 2021;238(11):3325-3334.

53.	Brose LS, McDermott MS, McNeill A. Heated Tobacco Products and Nicotine Pouches: A Survey of People with Experience of Smoking and/or Vaping in the UK. *Int J Environ Res Public Health.* 2021;18(16).

54.	East N, Bishop E, Breheny D, Gaca M, Thorne D. A screening approach for the evaluation of tobacco-free 'modern oral' nicotine products using Real Time Cell Analysis. *Toxicol Rep.* 2021;8:481-488.

55.	Marynak KL, Wang X, Borowiecki M, et al. Nicotine Pouch Unit Sales in the US, 2016-2020. *Jama.* 2021;326(6):566-568.

56.	Havermans A, Pennings JLA, Hegger I, et al. Awareness, use and perceptions of cigarillos, heated tobacco products and nicotine pouches: A survey among Dutch adolescents and adults. *Drug Alcohol Depend.* 2021;229(Pt B):109136.

57.	Li L, Borland R, Cummings KM, et al. Patterns of Non-Cigarette Tobacco and Nicotine Use Among Current Cigarette Smokers and Recent Quitters: Findings From the 2020 ITC Four Country Smoking and Vaping Survey. *Nicotine Tob Res.* 2021;23(9):1611-1616.

58.	Hrywna M, Gonsalves NJ, Delnevo CD, Wackowski OA. Nicotine pouch product awareness, interest and ever use among US adults who smoke, 2021. *Tob Control.* 2022.

59.	Czaplicki L, Patel M, Rahman B, Yoon S, Schillo B, Rose SW. Oral nicotine marketing claims in direct-mail advertising. *Tob Control.* 2022;31(5):663-666.

60.	Lunell E, Fagerström K, Hughes J, Pendrill R. Pharmacokinetic Comparison of a Novel Non-tobacco-Based Nicotine Pouch (ZYN) With Conventional, Tobacco-Based Swedish Snus and American Moist Snuff. *Nicotine Tob Res.* 2020;22(10):1757-1763.

61.	Plurphanswat N, Hughes JR, Fagerström K, Rodu B. Initial Information on a Novel Nicotine Product. *Am J Addict.* 2020;29(4):279-286.

62.	Bishop E, East N, Bozhilova S, et al. An approach for the extract generation and toxicological assessment of tobacco-free 'modern' oral nicotine pouches. *FOOD AND CHEMICAL TOXICOLOGY.* 2020;145.

63.	Akhlaque G, Siddiqui MMU, Iqbal R, Vancy AA, Khan JA, Naz S. Marketing Strategies and Consumer and Sellers Perception About Oral Nicotine Pouches in Karachi, Pakistan. *Tob Use Insights.* 2024;17:1179173x241308145.

64.	Avila JC, Maglalang DD, Nollen NL, et al. Using Pod Based e-Cigarettes and Nicotine Pouches to Reduce Harm for Adults With Low Socioeconomic Status Who Smoke: A Pilot Randomized Controlled Trial. *Nicotine & tobacco research : official journal of the Society for Research on Nicotine and Tobacco.* 2024;26(9):1150-1158.

65.	Becker E, McCaffrey S, Lewis J, Vansickel A, Larson E, Sarkar M. Characterization of Ad Libitum Use Behavior of On! Nicotine Pouches. *Am J Health Behav.* 2023;47(3):428-449.

66.	Brierley MEE, Li RZ, Jongenelis M. Correlates of Susceptibility to Nicotine Pouch Use Among Australian Adolescents and Younger Adults. *Nicotine & Tobacco Research.* 2025.

67.	Brose L, Bunce L, Cheeseman H. Prevalence of Nicotine Pouch Use Among Youth and Adults in Great Britain-Analysis of Cross-Sectional, Nationally Representative Surveys. *Nicotine & tobacco research : official journal of the Society for Research on Nicotine and Tobacco.* 2025.

68.	Campbell C, Feehan M, Kanitscheider C, Makena PS, Cai J, Baxter SA. Designing Studies to Inform Tobacco Harm Reduction: Learnings From an Oral Nicotine Pouch Actual Use Pilot Study. *JMIR Form Res.* 2022;6(8):e37573.

69.	Clark SA, Baler G, Jarman KL, Byron MJ, Goldstein AO, Ranney LM. Qualitative perspective on nicotine pouches from adults who smoke cigarettes in North Carolina. *Tob Control.* 2025;34(3):378-381.

70.	Czaplicki L, Tfayli D, Spindle TR, et al. Content analysis of marketing features in US nicotine pouch ads from 2021 to 2023. *Tobacco Control.* 2024.

71.	Dai HD, Leventhal AM. Prevalence of Nicotine Pouch Use Among US Adults. *JAMA.* 2024;332(9):755-757.

72.	Freeland R, Battista D, Polster M, Chae R, Dianno N, Frost-Pineda K. Purchase Intent and Product Appeal of Velo Nicotine Pouches Among Current Tobacco Users and Nonusers of Tobacco. *Contributions to Tobacco & Nicotine Research.* 2023;32:66-76.

73.	Giovenco DP, Spillane TE, Lee HM, et al. Retail availability of oral nicotine pouches in 3 US cities: A geographic analysis. *Nicotine & tobacco research : official journal of the Society for Research on Nicotine and Tobacco.* 2025.

74.	Hanewinkel R, Hansen J. [Use of nicotine pouches in childhood and adolescence]. *Laryngorhinootologie.* 2025;104(8):505-512.

75.	Jabba SV, Silinski P, Yang AY, Ouyang W, Jordt SE. Artificial Sweeteners in US-Marketed Oral Nicotine Pouch Products: Correlation with Nicotine Contents and Effects on Product Preference. *Nicotine & tobacco research : official journal of the Society for Research on Nicotine and Tobacco.* 2024.

76.	Jackson SE, Brown J, Notley C, Shahab L, Cox S. Characterising smoking and nicotine use behaviours among women of reproductive age: a 10-year population study in England. *BMC Med.* 2024;22(1):99.

77.	Jackson SE, Brown J, Buss V, Shahab L. Prevalence of Popular Smoking Cessation Aids in England and Associations With Quit Success. *JAMA Netw Open.* 2025;8(1):e2454962.

78.	Jamal A, Park-Lee E, Birdsey J, et al. Tobacco Product Use Among Middle and High School Students - National Youth Tobacco Survey, United States, 2024. *MMWR Morbidity and mortality weekly report.* 2024;73(41):917-924.

79.	Jankowski M, Rees VW. Awareness and use of nicotine pouches in a nationwide sample of adults in Poland. *Tob Induc Dis.* 2024;22.

80.	Jongenelis MI, Brierley ME, Li R. Patterns of nicotine pouch use among young Australians. *Drug Alcohol Depend.* 2024;264:112428.

81.	Kanobe MN, Powell CY, Patrudu M, et al. Randomized crossover clinical studies to assess abuse liability and nicotine pharmacokinetics of Velo Oral Nicotine pouches. *Front Pharmacol.* 2025;16:1547073.

82.	Keller-Hamilton B, Curran H, Alalwan M, et al. Evaluating the Role of Nicotine Stereoisomer on Nicotine Pouch Abuse Liability: A Randomized Crossover Trial. *Nicotine Tob Res.* 2025;27(4):658-665.

83.	Keyser BM, Leverette R, McRae R, et al. In vitro toxicological evaluation of pouched portioned oral nicotine products. *Front Toxicol.* 2024;6:1452274.

84.	Liu J, Edmiston JS, Wang J, et al. Oral Health Effects Among Adults Switching from Cigarettes to on!® Nicotine Pouches Compared to Those Who Continue Smoking. *Oral Health Prev Dent.* 2025;23:189-201.

85.	Liu J, Halpern-Felsher B. Popularity of Zyn and other nicotine pouch brands among U.S. adolescents, young adults, and adults in 2021. *Prev Med.* 2024;185:108068.

86.	Liu J, McCauley D, Gaiha SM, Halpern-Felsher B. Perceptions of Harm and Addictiveness for Nicotine Products, THC e-Cigarettes, and e-Cigarettes with Other Ingredients Among Adolescents, Young Adults, and Adults. *Substance use & misuse.* 2024;59(14):2126-2136.

87.	Mallock-Ohnesorg N, Rabenstein A, Stoll Y, et al. Small pouches, but high nicotine doses-nicotine delivery and acute effects after use of tobacco-free nicotine pouches. *Front Pharmacol.* 2024;15:1392027.

88.	McEwan M, Haswell L, Baxter-Wright S, Meichanetzidis F, Jin T, Hardie G. Plasma Nicotine Pharmacokinetics of Oral Nicotine Pouches Across Varying Flavours and Nicotine Content *. *Contributions to Tobacco & Nicotine Research.* 2023;32:130-139.

89.	Mohr T, Probst E, Idel C, et al. Different Influence Pattern of Conventional and Alternative Sources of Smoking on Adhesion Molecules and Cytokine Secretion in THP-1 Monocytes. *Anticancer Res.* 2024;44(4):1455-1464.

90.	Munawar S, Faheem M, Bilal M, Akram A, Anwar H, Jamil Y. Elemental Analysis and Classification of Nicotine Pouches Using Machine Learning Assisted Laser Induced Breakdown Spectroscopy. *Arabian Journal for Science and Engineering.* 2025;50(1):489-506.

91.	Nawaz MT, Hameed AN, Yasin F, et al. Knowledge, attitudes, practices, and prevalence of nicotine pouches in Pakistan: where do we stand? *International Journal Of Community Medicine And Public Health.* 2023;10:2037-2046.

92.	O'Hagan LA. A taste of Nordic freedom: The problematic marketing of nicotine pouches in the United Kingdom. *Nordisk Alkohol Nark.* 2024;41(6):574-598.

93.	Ozga JE, Cornacchione Ross J, Hrywna M, Sharma A, Ling PM, Stanton CA. Oral nicotine product marketing expenditures in the USA from 2016 to 2023: trends over time by brand and targeted media outlets. *Tob Control.* 2025;34(3):382-385.

94.	Park-Lee E, Jamal A, Cowan H, et al. Notes from the Field: E-Cigarette and Nicotine Pouch Use Among Middle and High School Students - United States, 2024. *MMWR Morbidity and mortality weekly report.* 2024;73(35):774-778.

95.	Peraza N, Han DH, Whaley RC, et al. Appeal and Sensory Characteristics of Oral Nicotine Products in Young Adults Who Vape E-Cigarettes. *Nicotine & tobacco research : official journal of the Society for Research on Nicotine and Tobacco.* 2024.

96.	Phan L, Zarei K, Chen-Sankey J, et al. Exposure to nicotine pouch marketing and nicotine pouch experimentation among U.S. adults who use commercial tobacco. *Preventive medicine reports.* 2024;46:102868.

97.	Prasad K, Edward L. Brief Survey of Current Oral Tobacco and Nicotine Product Use in Sweden and Denmark. *Contributions to Tobacco & Nicotine Research.* 2025;34:34-39.

98.	Prasad K, Shetty M, Kanitscheider C, Szentes B, Nassar R, Edward L. Assessing consumer use and behaviour patterns of oral nicotine pouches in a multi-country study. *International Journal of Scientific Reports.* 2022;8:173.

99.	Reimann H, Berger M, Eckert E, Merches K, Börnke F. Beyond smoking: Risk assessment of nicotine in pouches. *Toxicol Rep.* 2024;13:101779.

100.	Renard K, Nishihara D, Nilsson J, Larroque S, Martinez J, Giles L. Characterization of the nicotine uptake and safety of Nordic spirit tobacco-free oral nicotine pouches: A randomized cross-over study. *Psychopharmacology (Berl).* 2025;242(4):855-865.

101.	Reyes-Guzman CM, Baker L, Goss-Holmes H, Bloch MH. Patterns of Emerging Tobacco Product Use Among U.S. Adults, 2019-2022. *Am J Prev Med.* 2025;68(3):616-621.

102.	Ross JC, Kowitt SD, Rubenstein D, et al. Prevalence and correlates of flavored novel oral nicotine product use among a national sample of youth. *Addictive Behaviors.* 2024;152.

103.	Ruokolainen O, Ollila H, Haerkaenen T. Nicotine pouch use by sex, school type and tobacco product use among Finnish adolescents during the 2023 change in regulatory scheme with deregulated sales. *Addiction (Abingdon, England).* 2024;119(11):2023-2030.

104.	Shah NA, Li Z, McMann T, et al. Identification and Characterization of Synthetic Nicotine Product Promotion and Sales on Instagram Using Natural Language Processing. *Nicotine & tobacco research : official journal of the Society for Research on Nicotine and Tobacco.* 2024;26(5):580-588.

105.	Sharma A, Belton A, Ozga JE, et al. Marketing strategies in business-to-business advertisements for oral nicotine products. *Tobacco Control.* 2024.

106.	Silver N, Kucherlapaty P, Kierstead E, Schillo B. Objective Characteristics and Subjective Responses of 18-24-Year-Olds to U.S. nationally Televised Tobacco Advertisements. *Substance use & misuse.* 2024;59(7):1115-1125.

107.	Tran H, Tyx RE, Valentin L, Mahoney M, Stanfill S, Watson CH. Total and unprotonated (freebase) nicotine content in new types of oral 'tobacco-free' nicotine products. *Tobacco Control.* 2024.

108.	Watts C, McGill B, Rose S, et al. 'It'll save your lungs': early insights into nicotine pouch use and perceptions among young Australians. *Health Promot Int.* 2024;39(6).

109.	Aldeek F, Wagner K, Haase V, Sublette E, Lopez V. Analysis and Toxicological Evaluation of Nicotine Degradants and Impurities in Oral Tobacco Products: A Case Study of on! PLUS Nicotine Pouches. *ACS Omega.* 2025;10(6):6267-6278.

110.	Alkharaan H, Alrubayyi A, Kariri M, et al. Investigating oral nicotine pouch use among adults in Riyadh, Saudi Arabia: prevalence, awareness, susceptibility, and associated symptoms. *Front Public Health.* 2025;13:1607656.

111.	Alwafi H, Naser AY, Alharbi R, et al. Prevalence, safety, and role of nicotine pouches in smoking cessation among smokers and the public in Saudi Arabia. *Sci Rep.* 2025;15(1):29758.

112.	Azzopardi D, Brown E, Meichanetzidis F, et al. A Randomized Crossover Clinical Study to Assess the Effect of Oral Nicotine Pouches Used for Different Durations on Plasma Nicotine Pharmacokinetics in Healthy Oral Pouch Consumers. *J Clin Pharmacol.* 2025.

113.	Battista K, Piché-Ayotte M, Haddad S, Turcotte-Tremblay AM, Leatherdale ST, Bélanger RE. Emerging use of oral nicotine pouches among Canadian adolescents: Findings from the COMPASS-Quebec study. *Can J Public Health.* 2025.

114.	Brierley ME, Li R, Jongenelis MI. Perceptions of nicotine pouches among Australian adolescents, young adults, and adults. *Addict Behav.* 2025;170:108415.

115.	Cohen JE, Ibrahim M, Saraf S, et al. Velo (BAT) buffet of oral nicotine pouches in Pakistan: varying brand variant levels of nicotine, menthol and other flavour chemicals. *Tob Control.* 2025.

116.	Dai HD, Young B. Biomarkers of Exposure to Tobacco-Related Toxicants among Adult Nicotine Pouch Users. *Nicotine Tob Res.* 2025.

117.	Delnevo CD, Tomaino M, Hrywna M, Bover Manderski MT. Patterns of Nicotine Pouch Use Among Adults in the US, 2022-2023. *JAMA Netw Open.* 2025;8(9):e2531155.

118.	Elsokkary EM, Alsabhan FA, Alyahya AA, et al. Exploring the effect of nicotine pouches on users' health in Saudi Arabia: A cross-sectional study. *Tob Induc Dis.* 2025;23.

119.	Eversole A, Imran R, Gaitan N, et al. Physiological and subjective effects of an oral nicotine pouch in people who use smokeless tobacco. *Exp Clin Psychopharmacol.* 2025;33(5):483-493.

120.	Farzeen M, Ansari S, Khan A, et al. Exposure, access, susceptibility to and use of nicotine and tobacco products among 10-16 year-olds: National cross-sectional survey of 14,232 in-school and out-of-school children in Pakistan. *Nicotine Tob Res.* 2025.

121.	Farzeen M, Moodie C, Khan Z, et al. The availability and marketing of nicotine pouches at tobacco-selling points-of-sale in Pakistan. *Nicotine Tob Res.* 2025.

122.	Feizy S, Mattingly DT, Ickes M, et al. Tobacco marketing exposure and lifetime and current nicotine pouch use among US youth, 2022. *Prev Med.* 2025;197:108322.

123.	Fucito LM, Baldassarri SR, Wu R, et al. Effects of oral nicotine pouches on cigarette smoking behaviour and tobacco harm exposure: a randomised pilot trial in adults. *Tob Control.* 2025.

124.	Han DH, Harlow AF, Miech RA, et al. Nicotine Pouch and E-Cigarette Use and Co-Use Among US Youths in 2023 and 2024. *JAMA Netw Open.* 2025;8(4):e256739.

125.	Harlow AF, Han DH, Cho J, et al. Oral Nicotine Product Use and Vaping Progression Among Adolescents. *Pediatrics.* 2025;155(6).

126.	He Y, Zhang Z, Keller-Hamilton B, et al. Trends of oral nicotine pouch prices and sales by product characteristics in the USA, 2021-2024. *Tob Control.* 2025.

127.	Keller-Hamilton B, Curran H, Atkinson L, et al. Examining the role of freebase nicotine in the harm reduction potential of oral nicotine pouches versus moist snuff: A randomized crossover trial. *Addiction.* 2025.

128.	Lee J, Merenda F, Weinberger AH. Receiving coupons and discounts for nicotine pouches is associated with current use of nicotine pouches among United States adults: Results from the population assessment of tobacco and health (PATH) study wave 7 (2022-2023). *Drug Alcohol Depend Rep.* 2025;16:100370.

129.	Lyu JC, Ozga JE, Stanton CA, et al. Advertising the leading US nicotine pouch brand: a content analysis of ZYN advertisements from 2019 to 2023. *Tob Control.* 2025.

130.	Page MK, Leigh NJ, Block AC, et al. Comparison of Nicotine and Selected Flavorings Content Between Tobacco-Free and Tobacco-Containing Oral Pouches. *Nicotine Tob Res.* 2025.

131.	Palmer AM, Smith TT, Chen AA, Rojewski AM, Carpenter MJ, Toll BA. Nicotine Pouch Use in Youths and Adults Who Use Cigarettes, E-Cigarettes, and Smokeless Tobacco. *JAMA Netw Open.* 2025;8(5):e2511630.

132.	Taylor E, Ebdon M, Nottage M, et al. Investigating the Appeal of Nicotine Pouch Packaging, Flavour and Nicotine Descriptors among Adults in the UK: An Online Experiment. *Nicotine Tob Res.* 2025.

133.	Turner WG, Natarajan B, Bignell EM, et al. Characterizing Oral Synthetic Nicotine Pouch Use in Post-Secondary Students. *J Community Health.* 2025.

134.	Li CX, Wang PC, Feliu A, et al. Advertising exposure and the appeal of emerging nicotine-containing products among people who have never used nicotine: findings from the Eurobarometer 2023. *Tob Control.* 2025.

135.	Ruokolainen O, Ollila H, Karjalainen K. Correlates of the use of different nicotine strengths in smokeless tobacco (snus) and oral nicotine pouches among the Finnish general population. *Tob Control.* 2025.
