# Supplementary material for: Oral nicotine pouches and public health: evidence from an updated scoping review
Source: Nicotine Tob Res. Author manuscript; Available in PMC 2026 Oct 13. (PMC13660210; doi:10.1093/ntr/ntag203)
Supplement: Supplementary file 1 [file NIHMS2214286-supplement-Supplementary_Material.zip › Supplementary Table S2. Prevalence estimates of lifetime and current ONP use by survey year, country, and tobacco use status_R&R_CLEAN.docx]

**Oral Nicotine Pouches and Public Health: Evidence from an Updated Scoping Review**

Supplementary Table S2. Prevalence estimates of lifetime and current ONP use in youth and adults by survey year, country, and tobacco use status.

| Author (Survey Year) | Country | Study Design | Population | Tobacco use status | Lifetime ONP use, % | Current ONP use, % |
| --- | --- | --- | --- | --- | --- | --- |
| *****Jamal et al. (2024)** | US | Nationally representative school-based cross-sectional survey (NYTS) | Middle and high school students (grades 6 through 12), (n=29,861) | Not specific- general population | 3.5  (ever used even once or twice) | 1.8  (past 30-day use) |
| *****Park-Lee et al. (2024)** | US | Nationally representative school-based cross-sectional survey (NYTS) | Middle and high school students (grades 6 through 12), (n=29,861) | Not specific- general population | N/A | 1.8  (past 30-day use) |
| ***Han et al. (2023; 2024)** | US | Nationally representative school-based cross-sectional survey (MTF) | 10^th^ and 12th-grade school students (n=10,146) | Not specific- general population | 3 (2023); 5.4 (2024)  (ever used in a lifetime) | 1.3 (2023); 2.6 (2024)  (past 30-day use) |
| **Birdsey et al. (2023)** | US | Nationally representative school-based cross-sectional survey (NYTS) | Middle and high school students (n=22,069) | Not specific- general population | 2.3  (ever used even once or twice) | 1.5  (past 30-day use) |
| ***Palmer et al. (2022-2023)** | US | Nationally representative cross-sectional survey (PATH 7) | Youth ages12-17, (n=10, 632) | Not specific- general population | 0.9  (ever used in a lifetime) | 0.2  (past 30-day use) |
| **Park-Lee et al. (2022)** | US | Nationally representative school-based cross-sectional survey (NYTS) | Middle and high school students (N=2.51 million) | Not specific- general population | 2.3  (ever used even once or twice) | 1.1  (past 30-day use) |
| ***Feizy et al. (2022)** | US | Nationally representative school-based cross-sectional survey (NYTS) | Middle and high-school students (n=26,860) | Not specific- general population | 2.2  (ever used even once or twice) | 1.0  (past 30-day use) |
| *Ross et al. (2022) | US | National online survey | Youth ages 15-20 (n=680) | Ever or current users or those susceptible to use of little filtered cigars or cigarillos (LCCs). | N/A | 3.1  (past 30-day use) |
| Tosakoon et al. (2022) | US (Atlanta, Boston, Minneapolis, Oklahoma City, San Diego, and Seattle). | Cross-sectional survey | Youth and young adults ages 18–34 (n=942) | Oversampling of cigarette/ENDS users | 9.8  (ever used in a lifetime) | 2.2  (past 30-day use) |
| *Silver et al. (2021-2022) | US | Cross-sectional survey | Young adults ages 18-24 (n=1450) | Oversampled with past 30-day cigarette users | N/A | 24  (past 30-day use) |
| Patel et al. (2021-2022) | US | National cross-sectional survey | Youth and young adults ages 15-24 (n = 7,832) | Not specific- general population | 16  (ever used in a lifetime) | 12  (past 30-day use) |
| Gaiha et al. (2021) | US | Cross-sectional survey of a convenience sample | Adolescents, young adults, and adults. Ages 13–40  years (n = 6,131) | Not specific- general population | 17.9  (ever used in a lifetime) | 10.5  (past 30-day use) |
| **Kramer et al. (2021)** | US | Nationally representative school-based cross-sectional survey (NYTS) | Middle and high school students (n = 20, 413) | Not specific- general population | 1.9  (ever used even once or twice) | 0.8  (past 30-day use) |
| **Gentzke et al. (2021)** | US | Nationally representative school-based cross-sectional survey (NYTS) | Middle and high school students (N=27.41 million) | Not specific- general population | 1.9  (ever used even once or twice) | 0.8  (past 30-day use) |
| Harlow et al. (2021) | US, Southern California | Cross-sectional school-based survey | 9^th^ and 10^th^ graders (n=3,516) | Not specific- general population | 0.6  (ever used in a lifetime) | N/A |
| ***Palmer et al. (2022-2023)** | US | Nationally representative cross-sectional survey (PATH 7) | Adults ≥18 years (n=29,754) | Not specific- general population | 3.3  (ever used in a lifetime) | 0.8  (past 30-day use) |
| ***Delnevo et al. (2022-2023)** | US | Nationally-representative cross-sectional survey (TUS-CPS) | Adults 18+ years (n=110, 084) | Not specific- general population | 2.7  (ever used in a lifetime) | 0.4  (current use of nicotine pouches daily or on  some days) |
| ***Dai &Leventhal. (2022)** | US | Nationally-representative cross-sectional survey (TUS-CPS) | Adults 18+ years (n=39,558) | Not specific- general population | 2.9  (ever used in a lifetime) | 0.4  (past 30-day use) |
| ***Reyes-Guzman et al. (2022)** | US | Nationally-representative cross-sectional survey (TUS-CPS) | Adults 18 + years (n=40,000) | Not specific- general population | 2.9  (ever used in a lifetime) | 0.4  (current use of nicotine pouches daily or on  some days) |
| ***Phan et al. (2021)** | US | Nationally representative cross-sectional survey | Adults 21+ (n =1,700), with oversampling of self-identified Asian/Asian Americans and Black/African Americans | Current and former commercial tobacco users | 17.4  (used it before but not currently, and currently using it some days or every day) | N/A |
| **Schneller et al. (2019-2021)** | US | Nationally-representative cross-sectional survey (ITC Youth 2019, 2020,2021) | Adolescents ages 16-19, Waves 3-5 | Not specific- general population | 3.5  (Aug, 2019)  4.0  (Feb, 2020)  3.8  (Aug, 2020)  4.8  (Feb, 2021)  4.1  (Aug, 2021)  (ever used in a lifetime) | 1.5  (Aug, 2019)  1.8  (Feb, 2020)  1.7  (Aug, 2020)  2.0  (Feb, 2021)  2.0  (Aug, 2021)  (past 30-day use) |
| Morean et al. (2021) | US | Cross-sectional survey of a convenience sample | Young adults ages 18–25 (n=609) | Oversampling of current tobacco users | 10.3  (ever used in a lifetime) | N/A |
| Morean et al. (2021)◊ | US | Cross-sectional survey of a convenience sample | Young adults ages  18-25 (n=630) | Oversampled current ENDS and other tobacco product users | 9.7  (ever used in a lifetime) | N/A |
| Couch et al. (2021) | US | National cross-sectional survey | Adolescents and young adults ages 14-20 (n=2,253) | ENDS users | 29  (ever used in a lifetime) | 11  (past 30-day use) |
| **Sparrock et al. (2021)** | US | Nationally representative cross-sectional survey | Adults ages ≥21 (n=1583). | Current and former tobacco users | 16.4  (ever used in a lifetime) | 3  (current use daily or on  some days) |
| **Hrywna et al. (2021)** | US | Nationally representative cross-sectional survey | Adults ages ≥18 years (n=1,018) | Current established smokers | 5.6  (ever used in a lifetime) | N/A |
| Felicione et al. (2020) | US | Population-based cross-sectional survey (ITC) | Adults ages ≥18 years (n=2,507) | Current and former smokers and ENDS users | 3  (ever used in a lifetime) | 0.9  (past 30-day use) |
| East et al. (2019) | US | Population-based cross-sectional survey (ITC Youth) | Adolescents ages 16-19 (n=11,838) | Not specific- general population | N/A | 1.5  (past 30-day use) |
| ***Jankowski & Rees. (2024)** | Poland | Nationally-representative cross-sectional survey | Adults ≥18 years (n=1,080) | Not specific- general population | 9.2  (ever used in a lifetime) | 4.3  (past 30-day use) |
| *Alwafi et al. (2024-2025) | Saudi Arabia | Cross-sectional survey | Adults ≥18 years (n=657) | Tobacco users | 21.2  (ever tried in a lifetime) | N/A |
| *****Alkharaan et al. (2024**)** | Saudi Arabia | Cross-sectional survey | Adults ≥18 (n=831) | Not specific- general population | 14.2  (ever used in a lifetime) | N/A |
| *Jongenelis et al. (2024) | Australia | Cross-sectional survey | Youth and adults ages 16–39 (n=1,598) | Not specific- general population | 26  (ever used in a lifetime) | 19  (past 30-day use) |
| *Ruokolainen et al. (2023) | Finland | Nationwide cross-sectional school-based survey | Youth ages 14-20 (n= 151,224) | Not specific- general population | N/A | 14.6  (use ‘daily’ or ‘every now and then’) |
| ***Ruokolainen et al. (2022)** | Finland | Nationally-representative cross-sectional survey | Youth and adults ages 15–69 (n=3,857), with younger age groups (15–39 years) oversampled | Not specific- general population | 14.4  (used ‘daily or almost daily’, ‘occasionally’, ‘used, but have since quit’, or ‘tried a couple of times’) | 3.7  (use ‘daily or almost  daily’ and ‘occasionally’) |
| *Brose et al. (2024) | Great Britain | Cross-sectional survey | Youth ages 11-18 (n=2,584) | Not specific- general population | 3.3  (tried nicotine pouches, but do not use them + tried and still use them) | 1.2  (tried nicotine pouches and still use them) |
| *Brose et al. (2024) | Great Britain | Cross-sectional survey | Adults ≥ 18 years (n=13,266) | Not specific- general population | 5.4  (tried nicotine pouches, but do not use them + tried and still use them) | 1  (tried nicotine pouches and still use them) |
| *****Taylor et al. (2024) | UK | Cross-sectional survey | Adults ≥ 18 years (n=2,967) | Not specific- general population | 13  (tried nicotine pouches, but do not use them + tried and still use them) | N/A |
| ***Jackson et al. (2023)** | England | Nationally representative cross-sectional survey | Adults ages ≥ 18 years (n=197,266) | Not specific- general population | N/A | 0.4  (definition of current use unclear) |
| **Tattan-Birch et al. (2020-2021)** | Great Britain (England, Scotland, and Wales) | Representative cross-sectional survey | Adults ages ≥18 years (n=25,698) | Not specific- general population | N/A | 0.32  (current use (further definition unclear)) |
| **Brose et al. (2019)** | UK | Representative cross-sectional survey | Adults ages ≥18 years (n=3,883) | Current or former smokers and/or ENDS users | 4.4  (use daily, less than daily but at least once a week, less than weekly but at least once a month, tried a few times, tried once) | 2.7  (use daily, less than daily but at least once a week, less than weekly but at least once a month) |
| East et al. (2019) | England | Population-based cross-sectional survey (ITC Youth) | Adolescents ages 16-19 (n=11,070) | Not specific- general population | N/A | <1.5  (past 30-day use) |
| *Hanewinkel & Hansen. (2022-2023) | Germany | Cross-sectional survey | School students ages 9-17 (n=12,655) | Not specific- general population | 5.4  (ever used in a lifetime) | N/A |
| ***Farzeen et al. (2023-2024)** | Pakistan | Nationally representative cross-sectional survey | 10–16-year-old children in school and out of school (n=14,232) | Not specific- general population | N/A | 3.4 among boys; 2.7 among girls  (past 30-day of VELO pouch) |
| *Nawaz et al. (2022) | Pakistan | Cross-sectional survey | Youth and adults ages 13-56 (n=1,749) | Not specific- general population | 17.2  (ever used) | 9.4  (current use (yes/no)) |
| **Havermans et al. (2020)** | Netherlands | Nationally representative cross-sectional survey | Adolescents ages 13-17 (n=406) | Not specific- general population | 0.3  (current use or regular or occasional use in the past) | 0  (current use either daily, once a week, once a month, or less than monthly, but occasionally) |
| **Havermans et al. (2020)** | Netherlands | Nationally representative cross-sectional survey | Adults ages ≥18 years (n=5,399) | Not specific- general population | 0.6  (current use or regular or occasional use in the past) | 0.07  (current use either daily, once a week, once a month, or less than monthly, but occasionally) |
| Li et al. (2020) | England, Canada, Australia, US | Population-based cross-sectional survey (ITC) | Adults ages ≥18 years (n=10,296) | Current and former smokers | N/A | 1.1   (England)  0.9 (Canada)  0.8 (Australia)  0.8  (US)  (use monthly or at least ‘less than weekly’) |
| *Battista et al. (2024) | Canada (Quebec) | Cross-sectional survey | Students in grades 7-11 (n=13,914) | Not specific- general population | 3.7  (ever tried the product) | 2.6  (past 30-day use) |
| *Turner et al. (2024) | Canada | Cross-sectional survey | University or college students ≥18 years (n=452) | Not specific- general population | 27  (ever used in a lifetime) | 12  (past 30-day use) |
| East et al. (2019) | Canada | Population-based cross-sectional survey (ITC Youth) | Adolescents ages 16-19 (n=11,714) | Not specific- general population | N/A | <1.5  (past 30-day use) |
| ***Prasad & Edward. (2019)§** | Sweden/Denmark | Nationally representative cross-sectional survey | Adults ages 18–64 in Sweden (n = 7003) and Denmark (n = 4402) | Not specified | N/A | 1.4 (Sweden)  2.4 (Denmark)  (past 30-day use) |

*=Newly identified studies in the updated scoping review. Representative surveys are marked in bold. NYTS= National Youth Tobacco Survey. ITC= International Tobacco Control Policy Evaluation Project. TUS-CPS= Tobacco Use Supplement to the Current Population Survey. MTF= Monitoring The Future Survey. PATH= Population Assessment of Tobacco and Health Survey. ENDS=Electronic Nicotine Delivery Systems. ◊ Use of synthetic nicotine pouches (e.g., Niin, Fre, Rush and 2One brands). N/A= Not available. § Tobacco industry-funded study.
