# Supplementary material for: Oral nicotine pouches and public health: evidence from an updated scoping review
Source: Nicotine Tob Res. Author manuscript; Available in PMC 2026 Oct 13. (PMC13660210; doi:10.1093/ntr/ntag203)
Supplement: Supplementary file 1 [file NIHMS2214286-supplement-Supplementary_Material.zip › Supplementary Table S3. Demographic characteristics of lifetime and current ONP users.docx]

**Oral Nicotine Pouches and Public Health: Evidence from an Updated Scoping Review**

Supplementary Table S3. Demographic Characteristics of Lifetime and Current ONP Users by survey year, country, and tobacco use status.

| **Author (Survey Year)** | **Country** | **Study Design** | **Population** | **Tobacco use status** | **Lifetime use** | **Current use** |
| --- | --- | --- | --- | --- | --- | --- |
| ***Jamal et al. (2024)** | US | Nationally representative school-based cross-sectional survey (NYTS) | Middle and high school students (n=29,861) | Not specific- general population | ↑Males  ↑High school ages (vs. middle school)  ↑AI/AN, White and Multicultural students. | ↑Males  ↑High school ages (vs. middle school)  ↑AI/AN, White |
| ***Han et al. (2023-2024)** | US | Nationally representative cross-sectional survey (MTF) | 10 and 12 graders (n=10,146) | Not specific- general population | ↑Males  ↑12th grade  ↑ Rural  ↑Non-Hispanic White | ↑Males  ↑12th grade  ↑ Rural  ↑Non-Hispanic White |
| ***Palmer et al. (2022-2023)** | US | Nationally representative cross-sectional (PATH) | Youth age 12-17 (n=10,632) | Not specific- general population | ↑Males  ↑Older age (15-17)  ↑Non-Hispanic White  ↑Current and former smoking (vs. never), ↑Current and former SLT use (vs. never)  ↑Past year other oral nicotine product use  ↑Past year cannabis use  ↑Current ENDS use  ↑Former ENDS use | ↑Males  ↑Older age (15-17)  ↑Non-Hispanic White  ↑Current and former smoking (vs. never)  ↑Current and former SLT use (vs. never)  ↑Past year other oral nicotine product use  ↑Past year cannabis use  ↑Current ENDS use |
| **Birdsey et al. (2023)** | US | Nationally representative school-based cross-sectional survey (NYTS) | Middle and high school students (n=22,069) | Not specific- general population | ↑Male  ↑White  ↑High school (vs. middle school) | ↑Male  ↑High school (vs. middle school) |
| **Park-Lee et al. (2022)** | US | Nationally representative school-based cross-sectional survey (NYTS) | Middle and high school students (N=2.51 million) | Not specific- general population | ↑Male  ↑White  ↑High school (vs. middle school) | ↑Male  ↑High school (vs. middle school) |
| ***Feizy et al. (2022)** | US | Nationally representative school-based cross-sectional survey (NYTS) | Middle and high-school students (n=26,860) | Not specific- general population | ↑Self-reported past exposure to advertisements for tobacco products  ↑Lifetime SLT use (vs. never)  ↑Lifetime e-cigarette use (vs. never)  ↑Lifetime cigarette use (vs. never) | ↑Self-reported past exposure to advertisements for tobacco products  ↑Current SLT use (vs. non-current)  ↑Current e-cigarette use (vs. non-current)  ↑Current cigarette use (vs. non-current) |
| ***Palmer et al. (2022-2023)** | US | Nationally representative cross-sectional (PATH) | Adults ages ≥18 (n=29,754) | Not specific- general population | ↑Males  ↑Younger ages 18-64 (vs. 65+)  ↑Non-Hispanic White (vs. Black and Other races)  ↑Current and former smokers  ↑Current and former ENDS users  ↑Current and former SLT users  ↑Past year other oral product use  ↑Past year cannabis use  X Income  X Education | ↑Males  ↑Younger ages 18-64 (vs. 65+)  ↑Non-Hispanic White (vs. Black and Other races)  ↑Current and former smokers  ↑Current and former ENDS users  ↑Current and former SLT users  ↑Past year other oral product use  ↑Past year cannabis use  x Income  x Education |
| ***Lee et al.(2022-2023)** | US | Nationally representative cross-sectional (PATH) | Adults ages ≥18 (n = 27,712) | Not specific- general population | N/A | ↑Males  ↑Young ages 18-24 (vs. older)  ↑Non-Hispanic White (vs. Black and Other races)  ↑Current ENDS use  ↑Current SLT use  x Household income |
| ***Delnevo et al. (2022-2023)** | US | Nationally representative cross-sectional survey (TUS-CPS) | Adults ages ≥18 (n=110, 084) | Not specific- general population | ↑Males  ↑Non-Hispanic White  ↑Current smokers (vs. never)  ↑Current ENDS use (vs. never)  ↑Ever SLT use  ↑Quit smoking and ENDS use prior to 2019 and since 2019. | ↑Males  ↑Non-Hispanic White  ↑Current smokers  ↑Current ENDS use  ↑Ever SLT use  ↑Quit ENDS use since 2019 |
| ***Dai & Leventhal, 2022** | US | Nationally representative cross-sectional survey (TUS-CPS) | Adults ages ≥18 (n=39 558) | Not specific- general population | ↑Ages 18-64 (vs. 65+)  ↑Males  ↑Non-Hispanic Whites  ↑Current and former smoker  ↑Past year smoking quit attempt  ↑Intention to quit smoking in the next 6 months  ↓Non-daily current smoker  ↑ Higher education (vs. no high school)  x Household income | ↑Ages 18-64 (vs. 65+)  ↑Males  ↑Non-Hispanic Whites  ↑Past year smoking quit attempt  x Household income  x Education |
| ***Reyes-Guzman, 2022** | US | Nationally representative cross-sectional survey (TUC-CPS) | Adults ages ≥18 years (n=40,000) | Not specific-general population | ↑Ages 18−34 years (vs. 35+)  ↑Males  ↑Non-Hispanic Whites  ↑Lower educational level (vs. college) ↑Income ≥$75,000 (vs. less)  ↑Employed  ↑Living within metropolitan areas  ↑Nonpartnered | ↑Ages 18−34 years (vs. 35+)  ↑Males  ↑Non-Hispanic Whites  ↑Lower educational level (vs. college)  ↑Income ≥$75,000 (vs. less)  ↑Employed  ↑Living within metropolitan areas  ↑Nonpartnered |
| Patel et al. (2021-2022) | US | National cross-sectional survey | Youth and young adults ages 15-24 (n = 7,832) | Not specific- general population | **↑** Ages 21+ vs. younger  **↑** Male  **↑** Lower income | **↑** Ages 21+ vs. younger  **↑** Male  **↑** Lower income |
| **Kramer et al. (2021)** | US | Nationally representative school-based cross-sectional survey (NYTS 2021) | Middle and high school students (n = 20, 413) | Not specific- general population | ↑Male  ↑White  ↑High school (vs. middle school)  ↑Ever SLT use  ↑Current SLT use | ↑Male  ↑High school (vs. middle school)  ↑Current SLT use |
| *Harlow et al. (2021-2024) | US (Southern CA) | Longitudinal survey | 9th graders (n=703) | Past 6-month ENDS users | N/A | ↑Hispanic  ↑Parental education less than high school  ↑Current other tobacco use |
| **Schneller et al. (2019-2021)** | US | Nationally representative cross-sectional survey (ITC Youth 2019, 2020,2021) | Adolescents ages 16-19, Waves 3-5 | Not specific- general population | ↑Age18 vs. younger  ↑Male  ↑White  ↑Current SLT use  ↑Current ENDS use  ↑Current cigarette use | ↑Age18 vs. younger  ↑Male  ↑White  ↑Current SLT use  ↑Current ENDS use  ↑Current cigarette use |
| **Sparrock et al. (2021)** | US | Nationally representative cross-sectional survey | Adults ages ≥21 (n=1583). | Current and former tobacco users | **↑** Ages 18-30 vs. 61+  **↑**Ages 31-45 vs. 61+  **↑**Current SLT use vs. non-use | **↑**Ages 18-30 vs. 61+  **↑**Ages 31-45 vs. 61+  **↑**Current ENDS use  **↑**Current cigarette use  **↑**Current SLT use |
| Morean et al. (2021) | US | Cross-sectional survey of a convenience sample | Young adults ages 18–25 (n=609) | Oversampling of current tobacco users | **↑** Younger age  **↑** Lifetime ENDS use  **↑** Lifetime cigars/cigarillo use  **↑** Lifetime SLT use | N/A |
| Morean et al. (2021) ◊ | US | Cross-sectional survey of a convenience sample | Young adults ages 18-25 (n=630) | Oversampled current ENDS and other tobacco product users | **↑** Male  **↑** White  **↑** Lifetime SLT use  ˟ Lifetime ENDS, cigarette, cigar or hookah use. | N/A |
| **Hrywna et al. (2021)** | US | Nationally representative cross-sectional survey | Adults ages ≥18 years (n=1,018) | Current established smokers | **↑** Ages 18-44 vs. >=45  **↑** Lifetime use of SLT  **↑** Previous quit attempts of smoking with traditional methods (e.g., NRTs)  ↓ Higher vs. lower education  ˟ Sex  ˟ Ethnicity  ˟ Lifetime ENDS use | N/A |
| Felicione et al. (2020) | US | Population-based cross-sectional survey (ITC 2020) | Adults ages ≥18 years (n=2,507) | Current and former smokers and ENDS users | **↑**Age 18-24 vs. >=55,  **↑** Males  **↑** Current smokeless tobacco users vs. non users  **↓** Low and moderate education vs. high education  ˟ Ethnicity  ˟ Income | N/A |
| Plurphanswat et al. (2017-2018)§ | US | Cross-sectional survey | Adults ages ≥18 years (n=1,266) | Current ZYN users | N/A | Vast majority were:  men, white, with at least high school diploma with income more than $50 000, current SLT users and former tobacco users (mainly consisting of former dual cigarette‐SLT users) |
| *Alwafi et al. (2024-2025) | Saudi Arabia | Cross-sectional survey | Adults ≥18 years (n=657) | Tobacco product users | ↑Males  ↑Younger adults 18-30 ↑Students (vs. other occupational groups)  X Education level | N/A |
| *Alkharaan et al. (2024) | Saudi Arabia | Cross-sectional survey | Adults ≥18 years (n=831) | General population | ↑Males  ↑Younger ages 18-39 ↑Current cigarette use  ↑Current ENDS use | N/A |
| *Akhlaque et al. (2024) | Pakistan | Cross-sectional survey | Adult ages ≥18 (n=90) | Current ONP users | N/A | ↑Ages 18-25  ↑Current smokers  ↑Graduate degree |
| ***Jankowski &Rees (2024)** | Poland | Nationally representative cross-sectional survey | Adults ages ≥18 (n=1080) | General population | ↑Ages ≤60 years (vs. 60+) ↑Employed  ↑Current smokers  ↑Ever users of ENDS or heated tobacco  x Household income  x Gender | ↑Ages ≤60 years (vs. 60+)  ↑Employed  ↑Current smokers  ↑Current and ever users of ENDS or heated tobacco  x Household income  x Gender |
| *Jongenelis et al. (2024) | Australia | Cross-sectional survey | Youth and adults ages 16–39 years (n=1,598) | Not specific- general population | ↑Males  ↑Adults (vs. adolescents)  ↑Current and former tobacco users  ↑Current ENDS users | ↑Males  ↑Current and former tobacco users  ↑Current ENDS users |
| *Turner et al. (2024) | Australia | Cross-sectional survey | University or college students ages ≥18 older (n=452) | Not specific- general population | ↑Males  ↑White (vs. Asian)  ↑Ever cigarette, ENDS, cannabis use (vs. never)  X Financial status | N/A |
| *Hanewinkel &Hansen. (2022-2023) | Germany | Cross-sectional survey | Adolescents ages 9-17  (n=12,655) | Not specific- general population | ↑Males  ↑Ages 16-17 (vs. younger)  ↑Higher sensation seeking (vs. lower)  ↑Lower subjective social status (vs. higher)  ↑Co-use of other tobacco/nicotine products | N/A |
| *Rukolainen et al. (2023) | Finland | Cross-sectional survey | Youth ages 14-20 (n= 151,224) | Not specific- general population | N/A | ↑Males  ↑Daily use of other tobacco products |
| *Battista et al. (2024) | Canada (Quebec) | Cross-sectional survey | School students grades 7-11 (n=13,914) | Not specific- general population | N/A | ↑Males  ↑Older ages  ↑Youth with part-time jobs ↑Academically at-risk  ↑Current smoking  ↑Current ENDS use  ↑Lower risk perception  xFamily-level economic deprivation. |
| *Farzeen et al. (2023-2024) | Pakistan | Cross-sectional survey | 10–16-year-old children in school and out of school (n=14,232) | Not specific- general population | N/A | ↑Males  ↑Current other tobacco/nicotine products. |
| *Brose et al. (2024) | Great Britain | Cross-sectional survey | Youth ages 11-18 (n=2,584) | Not specific- general population | ↑Males  ↑Past year cannabis use  ↑Current and former smoker  ↑Current e-cigarette user | ↑Past year cannabis use  ↑Current smoker  ↑Current e-cigarette user |
| *Brose et al. (2024) | Great Britain | Cross-sectional survey | Adults ages ≥18 (n=13, 266) | Not specific- general population | ↑Ages 18-34 (vs. 55+)  ↑Males  ↑Mental Health Treatment  ↑Current and former smoking  ↑Current and former ENDS use  x Occupational grade  x Ethnicity  x Education | ↑Past year cannabis use  ↑Current smoking  ↑Current ENDS use |
| **Tattan-Birch et al. (2020-2021)** | Great Britain (England, Scotland, and Wales) | Representative cross-sectional survey | Adults ages ≥18 years (n=25,698) | Not specific- general population | N/A | **↑** Males  **↑** Ages 35-44 (vs.18-24)  **↑** Current smokers  **↑** Recent former smokers  **↑** Long-term (>1 year) former smokers (vs. never smokers)  **↑** Current ENDS and NRT users (vs. non-users) |
| **Brose et al. (2019)** | UK | Representative cross-sectional survey | Adults ages ≥18 years (n=3,883) | Current or former smokers and/or ENDS users | **↑** Age <45  **↑** Males  **↑** Higher education  **↑** Current smoking and vaping | N/A |

*Newly identified studies in the updated scoping review. Representative surveys are marked in bold. §Industry-funded. ◊ Use of synthetic nicotine pouches (e.g., Niin, Fre, Rush and 2One brands). MTF= Monitoring The Future Survey. NYTS= National Youth Tobacco Survey. TUS-CPS=Tobacco Use Supplement to the Current Population Survey. PATH- Population Assessment of Tobacco and Health Survey. SLT= Smokeless tobacco. ENDS= Electronic nicotine delivery systems. NRT= Nicotine replacement therapy. ↑Positive associations with ONP use. ↓Negative association with ONP use. ˟ No association with ONP use. N/A= Not available.
