# Supplementary material for: Oral nicotine pouches and public health: evidence from an updated scoping review
Source: Nicotine Tob Res. Author manuscript; Available in PMC 2026 Oct 13. (PMC13660210; doi:10.1093/ntr/ntag203)
Supplement: Supplementary file 1 [file NIHMS2214286-supplement-Supplementary_Material.zip › Supplementary Table S4. Pharmacokinetic profiles of ONPs compared to other tobacco and nicotine products.docx]

**Oral Nicotine Pouches and Public Health: Evidence from an Updated Scoping Review**

Supplementary Table 4. Pharmacokinetic profiles of ONPs compared to other tobacco/nicotine products.

| **Author, year** | **Funding** | **Sample** | **Test Product** | **Reference Product 1** | **Reference Product 2** |
| --- | --- | --- | --- | --- | --- |
| *Mallock-Ohnesorg et al., 2024 | Federal | Adult daily smokers (n=15) | Various brands, 6mg, 20mg, and 30 mg | Nicotine-free pouches:  No nicotine uptake | Cigarette:  Cmax (15.2 ng/ml) higher than for 6mg (2.8) and 20mg (7.1) ONPs, but lower than for 30mg (29.4) ONP.  Tmax (5 min) shorter than for 6mg (20 min), 20mg (15 min), and 30mg (15 min) ONPs.  AUC (0-240) 22.1 ng/mL × h) higher than for 6mg (4.9) and 20 mg (11.6) ONPs, but lower than for 30mg ONP (45.7) |
| *Keller-Hamilton et al., 2024 | Federal | Adult daily smokers (n=18) | Zyn (>99% S-nicotine [tobacco-derived]),  Niin and  Fre, 3mg, wintergreen | Racemic (synthetic) nicotine ONPs  Cmean lower than for tobacco-derived ZYN  Cmax (5.7 ng/mL) lower than for tobacco-derived ZYN (9.9 ng/mL)  Tmax (30 minutes) comparable between all ONPs |  |
| *Eversole et al., 2025 | Federal | Adult daily SLT users (n=23) | 2,4,8mg ONPs unflavored (original) | Own brand SLTs  First 30-min administration:  Cmean (10 ng/ml) higher than for 2mg (4.3 mg/nl) and 4mg (9.4 ng/ml) ONP, and lower than for 8mg (12.5 ng/ml) ONP.  Second 30-min administration:  Cmean (13 ng/ml) higher than for 2mg (4.8 mg/nl) and 4mg (10.4 ng/ml) ONP, and lower than for 8mg (14.3 ng/ml) ONP. |  |
| *Keller-Hamilton et al., 2025 | Federal | Adult daily moist snuff users, living in an Appalachian or rural Ohio county (n=62) | 6mg peppermint Rogue ONP (low FBN); 6 mg peppermint, Zyn ONP (high FBN) | Own-brand moist snuff  C_30min_ (12.3ng/ml) lower than for high FBN ONP (14.8 ng/ml); higher than for low FBN ONP (7.1 ng/ml).  C_max_ (13.7 ng/ml) lower than for high FBN ONP (15.1 ng/ml); higher than for low FBN ONP (7.5 ng/ml).  AUC_0-90_ (919 ng/ml) comparable to high FBN ONP (998 ng/ml); higher than for low FBN ONP (507 ng/ml).  T_max (41_ min) comparable to high FBN (36 min) and low FBN (42 min) ONPs. |  |
| *Kanobe et al., 2025 | BAT | Adult daily smokers (and dual smokers and SLT users) (study 1 n=41; study 2 n=43; study 3 n=36) | 4mg, 8mg, 12mg Velo menthol/mint; 4mg and 8mg Velo mini menthol/mint, and tobacco. | NRT gum  AUC_0-15,_ AUC_0-240,_ C_max_ lower than 4mg, 8mg, 12 mg ONP, 8mg mini ONP. Comparable to 4mg mini ONP.  T_max_ (35-40min) comparable to all ONPs. | Cigarette  AUC_0-15_ and C_max_ higher than for 4mg, 8mg, and 12 mg ONPs.  AUC_0-240_ higher than 4mg and 8mg ONP, comparable to 12mg ONP.  C_max_ higher than 4mg, 8mg, and 12 mg ONPs.  AUC_0-15_ higher than 4mg and 8mg mini ONPs  AUC_0-240_ higher or comparable to 4mg mini ONPs; lower than 8mg mini ONPs.  C_max_ higher than 4mg mini, comparable to 8mg mini ONPs.  T_max_ (5-7min) higher than all ONPs (35-40 min). |
| *Azzopardi et al., 2025 | BAT | Adult regular and daily consumers of snus or ONPs (n=35) | 11mg and 20mg Velo Freeze | C_max_ for 20mg ONP (≈33 ng/ml) higher than for 11mg ONP (≈22 ng/ml) at 30 minutes of use  AUC_0-240_ for 20mg ONP at 30 minutes of use (≈66 ng*h/ml) higher than for 11mg ONP (≈43 ng*h/ml)  T_max_ comparable between  the 11mg and 20mg ONPs when used for the same  durations. |  |
| *McEwan et al., 2023 | BAT | Adult current smokers (n=42) | 4mg and 7mg Velo of eight different flavors | C_max_ for 7mg ONP (≈16-18 ng/ml) higher than for 4mg ONP (≈11 ng/ml) at 45 minutes of use.  AUC_0-180_ for 7mg ONP (≈1800 ng*min/ml) higher than for 4mg ONP (≈1200 ng*min/ml)  T_max_ comparable between all ONPs (46 minutes) |  |
| *Renard et al., 2024 | JTI | Adult Swedish snus users aged 19–64 years (n=30) | 6mg-mint; 9mg-mint; 11.2mg-spearmint Nordic Spirit | Tobacco snus (11.2 mg/pouch)  C_max_ (8.18 ng/mL) lower than for 6 mg (10.92 ng/mL); 9 mg (13.81 ng/m (and 11.2 mg (17.33 ng/mL) ONPs.  AUC_0- 60_ (5.97 h*ng/mL) lower than for 6mg (8.56), 9mg (10.55) and 11.2mg (12.90 h*ng/mL).  T_max_ (45 min) higher than all ONPs (30-38 mins) | NRT gum  C_max_ (9.23 ng/mL) lower than for 6 mg (10.92 ng/mL); 9 mg (13.81 ng/m (and 11.2 mg (17.33 ng/mL) ONPs.  AUC_0- 60_ (6.45 h*ng/mL) lower than for 6mg (8.56), 9mg (10.55) and 11.2mg (12.90 h*ng/mL).  T_max_ (45 min) higher than all ONPs (30-38 mins) |
| Lunell et al., 2020 | Swedish Match | Adult snus users (n=29) | ZYN 3 mg, 6 mg, 8 mg | 8 mg General Snus:  AUC_inf_ (45.9 ng/mL h) higher than for ZYN 3 mg (32 ng/mL h)  AUC_inf_ lower than for ZYN 6 mg (57.7 ng/mL h)  C_max_ (10.6 ng/mL) higher than for ZYN 3 mg (7.7 ng/mL)  C_max_ lower than for ZYN 6 mg (14.7 ng/mL)  T_max_  (69 min) comparable to ZYN 3 mg (61 min) and ZYN 6 mg (61 min)  2 x 8 mg General Snus:  AUC_inf_ (70.3 ng/mL h) higher than for ZYN 8 mg (58.4 ng/mL h)  C_max_ (21.2 ng/mL) higher than for ZYN 8 mg (18.5 ng/mL) | 18 mg Longhorn moist snuff:  AUC_inf_, C_max_ and T_max_ not statistically different to ZYN 8 mg |
| Rensch et al., 2021 | Altria | Adult smokers (n=42) | Six flavors of ON! 4 mg | Own cigarette brand:  Higher C_max_ and shorter T_max_ compared to ONPs | N/A |
| Liu et al., 2022 | Altria | Adult dual cigarette and moist SLT users (n=30) | ON!1.5 mg, 2 mg, 3.5 mg, 4 mg, and 8 mg | Own cigarette brand:  C_max_ (12.2 ng/mL) higher than for 1.5 mg (3.2 ng/mL), 2 mg (4.6 ng/mL), 3.5 mg (7.1 ng/mL), and 4 mg (8.4 ng/mL) ON!  AUC_0-180_ (926 ng min/mL) comparable to 3.5 mg (699 ng min/mL) and 4 mg (796 ng min/mL) ON!  C_max_ and AUC_0-180_ lower than for 8 mg ON! (14.5 ng/mL and 1441 ng min/mL)  T_max_ (8.5 min) shorter than all ONPs (33-34 min) | Moist SLT:  C_max_ (9.8 ng/mL) higher than for 1.5 mg, 2 mg, and 3.5 mg ON!  C_max_ comparable to 4 mg ON!  AUC_0-180_ (1065 ng min/mL) higher than for 1.5 mg, 2 mg, 3.5 mg, and 4 mg ON!  C_max_ and AUC_0-180_ lower than for 8 mg ON!  T_max_ (34 min) comparable to all ONPs |
| McEwan et al., 2022 | BAT | Adult dual snus and cigarette users (n=35) | LYFT 10 mg | Combustible cigarette:  C_max_ (13.9 ng/mL) and AUC_0–6h_ (25.2 ng h/mL ) lower than for LYFT 10 mg (17.1 ng/mL; 53.7 ng h/mL)  T_max_ (7 min) shorter than all ONPs (60-65 min) | Other ONP brands (ZYN 10 mg, Nordic Spirit 9 mg, Skruf 8 mg, On! 6 mg):  C_max_ and AUC_0–6h_ lower for ZYN 10 mg (11.9 ng/mL; 35.8 ng h/mL) and Skruf 8 mg (13 ng/mL; 39 ng h/mL) than for LYFT 10 mg (17.1 ng/mL; 53.7 ng h/mL)  C_max_ and AUC_0–6h_ comparable for Nordic Spirit 9 mg (18.4 ng/mL; 52.8 ng h/mL), ON! 6 mg (17.5 ng/mL; 47 ng h/mL) and LYFT 10 mg (17.1 ng/mL; 53.7 ng h/mL) |
| Chapman et al., 2022 | Imperial Brands | Adult tobacco users (n=24) | ZoneX 5.8 mg and 10.1 mg | Combustible cigarette:  AUC_t_ (19.6 ng h/mL)and C_max_ (11.6 ng/mL) higher than for ONPs (12.2-18.4 ng h/mL; 5.2-7.9 ng/mL)  T_max_ (8.5 min) shorter than ONPs (22-26 min). | N/A |
| Azzopardi et al., 2022 | BAT | Adult smokers (n=36) | 4 mg ONPs (brand name n/a) | Nicorette® 4 mg nicotine gum:  AUC_t_ (14.3 ng h/mL), C_max_ (4.4 ng/mL)and T_max_ (0.83 h) lower than for ONP (30.6 ng h/mL; 8.5 ng/mL; 1 h). | Nicorette® 4 mg lozenge:  AUC_t_ (31.5 ng h/mL)C_max_ (8.3ng/mL) and T_max_ (1h) comparable to ONP |
| Keller-Hamilton et al., 2023 | Federal | Adult smokers (n=30) | ZYN 3 mg and 6 mg | Combustible cigarette:  Plasma nicotine levels at 30 mins lower than for ZYN 6mg and comparable to ZYN 3 mg. | N/A |

*Newly added studies. AUCinf - area under the plasma nicotine concentration-time curve from 0 to infinity. AUC0-180 - area under the plasma nicotine concentration-time curve from 0 to 180 minutes. AUC0-6h - area under the plasma nicotine concentration-time curve from 0 to 6 hours. AUCt area under the plasma concentration–time curve from time 0 to the time of the last sampling timepoint. Cmax- maximum observed plasma nicotine concentration. C_mean_ – mean plasma nicotine concentration. Tmax- time to maximum measured plasma nicotine concentration. FBN- free-base nicotine.
