# Supplementary material for: Oral nicotine pouches and public health: evidence from an updated scoping review
Source: Nicotine Tob Res. Author manuscript; Available in PMC 2026 Oct 13. (PMC13660210; doi:10.1093/ntr/ntag203)
Supplement: Supplementary file 1 [file NIHMS2214286-supplement-Supplementary_Material.zip › Supplementary File S1. Search strategy for the PubMed Database.docx]

Oral Nicotine Pouches and Public Health: Evidence from an Updated Scoping Review.

Supplementary File S1. Search strategy for the PubMed Database

| **PubMed** | | |
| --- | --- | --- |
| **Date Searched** | **Search String** | **Results** |
| January 1, 2023 through September 30, 2025 | (("VELO"[Title/Abstract] AND "pouch*"[Title/Abstract]) OR ("on"[Title/Abstract] AND "pouch*"[Title/Abstract]) OR "zyn"[Title/Abstract] OR ("nicotine"[Title/Abstract] AND "pouch*"[Title/Abstract]) OR ("tobacco-free"[Title/Abstract] AND "oral"[Title/Abstract] AND "pouch*"[Title/Abstract]) OR ("modern"[Title/Abstract] AND "oral"[Title/Abstract] AND "nicotine"[Title/Abstract] AND "product*"[Title/Abstract]) OR ("tobacco-free"[All Fields] AND ("lobeline"[MeSH Terms] OR "lobeline"[All Fields] OR "smokeless"[All Fields] OR ("tobacco, smokeless"[MeSH Terms] OR ("tobacco"[All Fields] AND "smokeless"[All Fields]) OR "smokeless tobacco"[All Fields] OR "snus"[All Fields]) OR ("tobacco, smokeless"[MeSH Terms] OR ("tobacco"[All Fields] AND "smokeless"[All Fields]) OR "smokeless tobacco"[All Fields] OR "snuff"[All Fields] OR "snuffs"[All Fields]))) OR ("nicotine-containing"[All Fields] AND "pouch*"[All Fields]) OR ("snus-like"[All Fields] AND "product*"[All Fields])) | 263 |
